# Coupling magnetic and plasmonic anisotropy in hybrid nanorods for mechanochromic responses

**DOI:** 10.1038/s41467-020-16678-8

**Published:** 2020-06-08

**Authors:** Zhiwei Li, Jianbo Jin, Fan Yang, Ningning Song, Yadong Yin

**Affiliations:** Department of Chemistry, University of California, Riverside, CA 92521 USA

**Keywords:** Metamaterials, Nanoparticles, Synthesis and processing, Nanophotonics and plasmonics, Nanosensors

## Abstract

Mechanochromic response is of great importance in designing bionic robot systems and colorimetric devices. Unfortunately, compared to mimicking motions of natural creatures, fabricating mechanochromic systems with programmable colorimetric responses remains challenging. Herein, we report the development of unconventional mechanochromic films based on hybrid nanorods integrated with magnetic and plasmonic anisotropy. Magnetic-plasmonic hybrid nanorods have been synthesized through a unique space-confined seed-mediated process, which represents an open platform for preparing next-generation complex nanostructures. By coupling magnetic and plasmonic anisotropy, the plasmonic excitation of the hybrid nanorods could be collectively regulated using magnetic fields. It facilitates convenient incorporation of the hybrid nanorods into polymer films with a well-controlled orientation and enables sensitive colorimetric changes in response to linear and angular motions. The combination of unique synthesis and convenient magnetic alignment provides an advanced approach for designing programmable mechanochromic devices with the desired precision, flexibility, and scalability.

## Abstract (web summary)

Programmable mechanochromic systems hold important roles in designing advanced bionic robots and colorimetric devices. Herein, the authors report the development of magnetic-plasmonic hybrid nanorods that can be integrated into thin polymer films with controlled orientations to display programmable mechanochromism.

## Introduction

Mechanochromic materials that exhibit reversible and predictable color changes have broad applications in mechanical sensors, security devices, bionic robots, and smart windows^1^. Most current systems rely on photonic structures^2–4^, fluorescence^5^, and plasmonic resonance^6,7^; they are limited to providing colorimetric responses to simple deformations under stretching and pressing and also lack the flexibility of large-scale programmable device fabrication. Therefore, engineering mechanochromic responses to complex perturbations remains a challenge, although it is highly desirable in many real-world applications that involve both linear and angular perturbations, such as rotation, bending, and twisting. The spatially differentiated photon-electron resonance of anisotropic plasmonic nanostructures^8,9^ offers excellent opportunities to achieve these colorimetric responses and has enabled a variety of fascinating applications such as diffraction-unlimited optics^10^, laser writing^11^, negative/zero-index metamaterials^12^, optical modulator^13^, and photothermal conversion^14,15^. Almost all of these explorations are, however, based on units that are either fabricated on solid substrates by advanced lithography and electrochemical self-assembly^16–19^ or carefully chosen from those chemically synthesized and then randomly deposited on substrates^20–22^. These methods produce anisotropic plasmonic nanostructures with fixed orientation relative to substrates and therefore lack flexibility for active tuning of plasmonic excitation for complex mechanochromic responses. Selective excitation of multiple plasmonic nanorods has been achieved via incorporation into liquid crystals (LCs)^23,24^, where orientational control could be realized by applying electric fields. Such a system may find applications in electrochromic displays^25,26^, but also shares the limitations of conventional LC devices. Through mechanical stretching of polymer matrices or masked metal evaporation, colloidal plasmonic nanoparticles were also made into oriented arrays to display polarization-dependent coloration^27–29^. Such systems, however, have limited flexibility in precise orientational control in the exact locations and matrices desired for fabricating complex mechanochromic devices. Magnetic–plasmonic hybrid nanostructures represent a class of smart nanomaterials for precise orientational control, and have been exploited in biomimetics, bioimaging, sensing, and information encryption^30–33^. They have been produced by co-assembly of plasmonic and magnetic nanomaterials^34–36^, and significant improvement is still desired in the dimensional control, structural stability, and the precision of alignment for designing mechanochromic films with predictable color changes^37^.

Here, we report the development of programmable mechanochromic films with precise colorimetric responses to a number of mechanical perturbations by preparing magnetic–plasmonic hybrid nanorods through an unconventional colloidal synthesis approach. The plasmonic nanorods are grown alongside the magnetic ones through a seed-mediated process confined within highly permeable polymer shells, producing compact hybrid nanorods with perfect structural alignment, coupled magnetic–plasmonic properties, and excellent colloidal stability. This versatile approach represents an open platform that allows the design of a wide range of high-quality complex nanostructures. Using Fe_3_O_4_/Au hybrid nanorods as the active components, we demonstrate that the coupled magnetic and plasmonic anisotropy can enable efficient control of their orientation and subsequently the plasmonic excitation through magnetic means, which is confirmed by simulation and analytical solution derived from bra-ket notation. Based on the clear orientation–excitation correlation and conventional lithography, we magnetically align hybrid nanorods along the desired directions in the defined locations of the polymer matrices and further develop plasmonic films with pre-designed mechanochromic responses under various mechanical perturbations.

## Results

### Synthesis of magnetic–plasmonic hybrid nanostructures

**Fig. 1:**
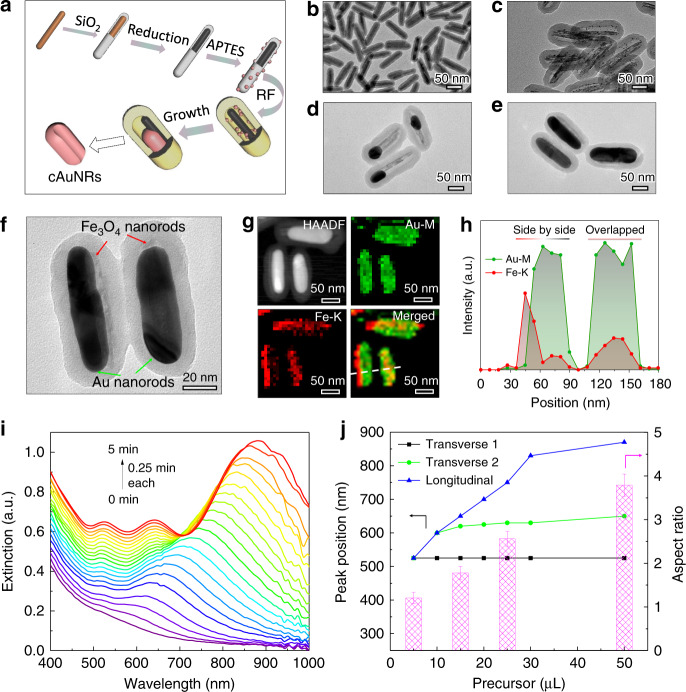
Synthesis and characterization of magnetic-plasmonic  hybrid nanostructures. **a** Scheme of the confined growth towards magnetic-plasmonic hybrid nanorods. In the last step of the scheme, Fe_3_O_4_ nanorod and RF shell are removed to clarify the concave structure of the Au nanorod. TEM images of nanorods after SiO_2_ coating (**b**), RF coating (**c**), seeded growth with 15 µL (**d**), 25 µL (**e**) of the precursor. **f** TEM image showing hybrid nanorods with two typical configurations (left: side by side; right: overlapped). **g** HAADF and EDS mapping images of the hybrid structures. **h** The cross-sectional line profile of element distribution. **i** The real-time extinction spectra of cAuNRs with a time interval of 15 s. **j** Dependence of peak positions of surface plasmonic resonance and aspect ratios of cAuNRs on the volume of the precursor. The reaction kinetics is controlled by adding different amounts of precursors as indicated. Error bars represent the standard deviations from the measurement of ten hybrids nanorods in TEM images.

The space-confined seed-mediated growth of magnetic-plasmonic hybrid nanorods is depicted in Fig. 1a. FeOOH nanorods (120 nm × 20 nm, Supplementary Fig. 1a) were synthesized by a high-temperature hydrolysis reaction^38,39^ and then reduced to Fe_3_O_4_ by a polyol process with the protection of a silica shell (Fig. 1b)^40,41^. The blocking temperature and the magnetic anisotropy constant of Fe_3_O_4_@SiO_2_ nanorods were found to be 190 K (below RT) and 1.8 kJ m^−3^ (one order lower than magnetocrystalline anisotropy constant), indicating superparamagnetism with a dominant shape anisotropy (Supplementary Fig. 1). After immobilizing Au seeds through electrostatic interaction^42^, the nanorods were overcoated with a layer of resorcinol phenol (RF) resin^43^, whose cross-linking was enhanced by further heating at 100 °C. Meanwhile, the silica interlayer was etched by a base, producing magnetic nanorods@voids@RF nanostructure with small Au seeds dispersed homogeneously within the RF shells (Fig. 1c).

The seeded growth of Au was carried out using our previously developed procedure^44^. Interestingly, once a small amount of HAuCl_4_ was added, only one isotropic Au nanoparticle formed within each RF shell (Supplementary Fig. 2a) due to Ostwald ripening, which involves the initial dissolution of smaller seeds by oxidative-etching by I^−^/O_2_ and then re-deposition to the larger seeds^45^. Further growth induced a unique concave structure along the long axis of Au nanorods (denoted as cAuNRs thereafter)^46^. Depending on the amount of added precursors, the seeds could grow progressively into cAuNRs with highly uniform size, shape, and perfect parallel alignment to the Fe_3_O_4_ nanorods (Supplementary Fig. 1d, e). Details of the unique concave structures are shown in Fig. 1f with two typical orientations (side-by-side and overlapped configurations) of cAuNRs. The hybrid nanostructure is further confirmed by element mapping (Fig. 1g) and energy-dispersive X-ray spectroscopy (EDS) analysis (Fig. 1h), with the latter clearly revealing a side-by-side (left) and overlapping (right) configurations. The growth was isotropic initially and then switched to anisotropic mode, with longitudinal plasmon modes appearing at a longer wavelength and red-shifting continuously to 880 nm (Fig. 1i). While both transverse and longitudinal peaks became stronger as the seeded growth proceeded, the peak due to the surface concave structure appeared at 630 nm (see Supplementary Discussion I). With more precursors, the growth became faster, resulting in cAuNRs with larger aspect ratios (Fig. 1j; Supplementary Fig. 2e). By manipulating the reaction kinetics, we could produce cAuNRs with different aspect ratios (up to 3.8) and correspondingly control their longitudinal resonance wavelengths.

### Bra-ket notation of plasmonic excitation

**Fig. 2:**
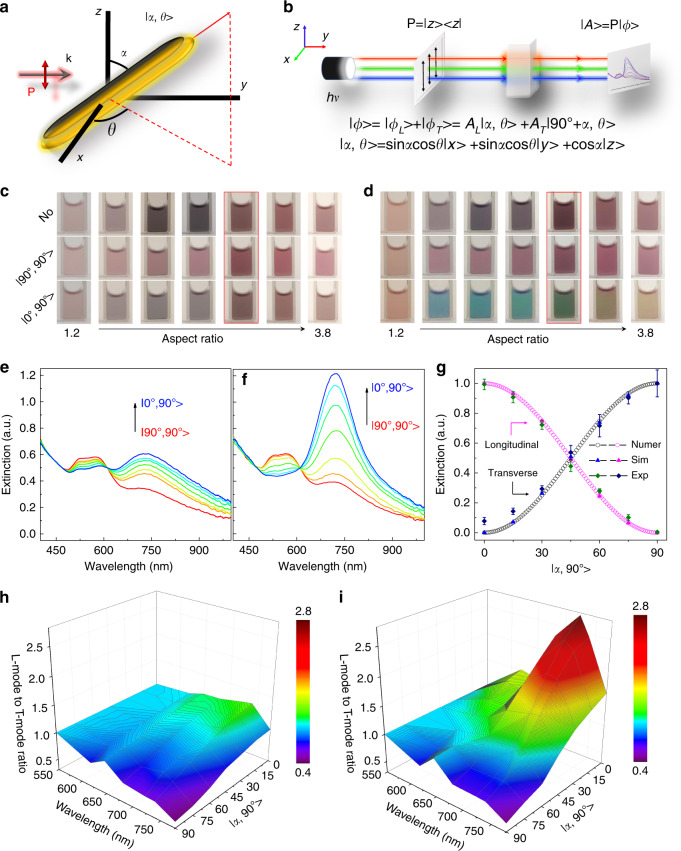
Optical tunability of colloidal cAuNRs. **a** Schematic illustration of cAuNRs under the orientational state |*α*, *Ɵ* > with respect to the polarization of light. **b** Tuning plasmonic extinction of cAuNRs under polarized light and the corresponding mathematical interpretation by bra-ket notation. **c**, **d** Digital images of cAuNRs dispersions under normal (**c**) and polarized light (**d**). In both **c** and **d**, the colloidal dispersions from left to right correspond to spectra in Supplementary Fig. 2f (from bottom to top). **e**, **f** Tuning the extinction of cAuNRs under normal (**e**) and polarized light (**f**) for samples of highlighted columns in (**c**) and (**d**), respectively. **e**, **f** share the same *y* axis. The spectra were measured with an angle step of 15^o^. **g** Correlation between the excitation modes and orientational states of cAuNRs, with the fine spectra tunability shown in **f**. The abbreviations, Numer, Sim, and Exp, represent numerical, simulation, and experimental results, respectively. Error bars represent the standard deviations from three experimental measurements. **h**, **i** Summary of L-mode to T-mode ratio of different dispersions in **c** and **d** achieved by varying *α* under normal (**h**) and polarized light (**i**). The azimuth angle, *Ɵ*, was set at 90^o^. P and k are the polarization and wave vector of the incident light, respectively.

To understand the plasmonic excitation of anisotropic nanostructures under linearly polarized light, we first derived the analytical equations of plasmonic excitation based on bra-ket notation. Figure 2a shows an arbitrary configuration of cAuNRs, whose orientation can be mathematically expressed by a ket, |α, *Ɵ* > . Under *z*-polarized light (Fig. 2b), the bra-ket notation of orientation state, ׀S > , of cAuNRs in Fig. 2a is expressed as *A*_L_׀α, *Ɵ* > + *A*_T_ | 90^o^+ α, *Ɵ* > , where the first and second terms determine the longitudinal and transverse excitation, respectively. Exciting plasmon resonance of cAuNRs under polarized light could be interpreted as polarizer operator (*P*, |*z* > < *z*|) operating on the corresponding ket (Supplementary Fig. 4):

1 $$\left| {{{A}} > = {P}} \right|\psi _{\mathrm{L}} > + {P}|\psi _{\mathrm{T}} > = {A}_{\mathrm{L}}{\mathrm{cos}}\alpha \left| {{z} > - {A}_{\mathrm{T}}{\mathrm{sin}}\alpha } \right|{z} > $$

where *A*_L_cosα and *A*_T_sinα represent longitudinal and transverse excitation coefficients, respectively. The resulted ket, |*z *> , indicates that the resonance happens along the *z* direction. Given an arbitrary orientation ket, the expectation value of excitation can be derived as follows by using the bra-ket theorem:

2 $$< {A}(\alpha ,\theta ) > = < \psi _{\mathrm{L}}\left| {P} \right|\psi _{\mathrm{L}} > + < \psi _{\mathrm{T}}\left| {P} \right|\psi _{\mathrm{T}} > = {I}_{\mathrm{L}}{\mathrm{cos}}^2\alpha + {I}_{\mathrm{T}}{\mathrm{sin}}^2\alpha$$

It predicts that the expectation value of excitation is only dependent on the azimuthal angle, *α* (see Supplementary Methods II). We used ratiometric data processing to quantify the correlation between excitation states and azimuthal angle, *α*, which helps to eliminate signal fluctuation and backgrounds:

3 $$\frac{{ < {E}_{\mathrm{L}}\left( {{\alpha }},{\theta} \right) > - < {E}_{\mathrm{L}}\left( {90^{\mathrm{o}},{\theta}} \right) > }}{{ < {E}_{\mathrm{L}}\left( {0^{\mathrm{o}},{\theta}} \right) > - < {E}_{\mathrm{L}}\left( {90^{\mathrm{o}},{\theta}} \right) > }} = {\mathrm{cos}}^2{\alpha}$$

4 $$\frac{{ < {E}_{\mathrm{T}}\left( {{\alpha }},{\theta} \right) > - < {E}_{\mathrm{T}}(0^{\mathrm{o}},{\theta}) > }}{{ < {E}_{\mathrm{T}}\left( {90^{\mathrm{o}},{\theta}} \right) > - < {E}_{\mathrm{T}}(0^{\mathrm{o}},{\theta}) > }} = {\mathrm{sin}}^2{\alpha}$$

where *E* is transverse or longitudinal extinction at given orientations.

### Tuning plasmonic excitation by magnetic fields

We first studied the orientation-dependent plasmonic excitation of cAuNRs by measuring the extinction of their colloidal dispersions in different magnetic fields. The perfect parallel alignment between cAuNRs and Fe_3_O_4_ nanorods, enabled by our unique synthesis, facilitated convenient magnetic control of the plasmon resonance of cAuNRs. Under an ordinary light (Fig. 2c), both transverse and longitudinal modes were excited (Supplementary Figs. 10, 11), and consequently, the solutions appeared gray. Under polarized light, selective excitation of transverse or longitudinal mode could be achieved by magnetically aligning cAuNRs to |90^o^, 90^o^ > and |0^o^, 90^o^ > , respectively (Fig. 2d; Supplementary Movies 1, 2). At |90^o^, 90^o^ > , only the transverse mode at 525 nm was excited, and the solution of cAuNRs with different ARs was red (middle panel in Fig. 2d). At |0^o^, 90^o^ > , the color of the solution turned from red to blue, green and finally yellow (bottom panel in Fig. 2d) due to the selective excitation of longitudinal modes and their continuous red-shift. The extinction spectra of cAuNRs with an aspect ratio of 2.5 are shown in Supplementary Fig. 2e, f under normal and polarized light, respectively. As shown in Fig. 2g and Supplementary Fig. 9, the dependence of plasmonic excitation on α from experimental measurements and simulation is consistent with the analytical solution of Eq. (2).

To quantitatively describe the color brightness of the dispersions, we calculated the ratio between longitudinal (E_L_) and transverse extinction (E_T_) as f(*α*) = E_L_(*α*)/E_T_(*α*). As summarized in Fig. 2h, the highest contrast under ordinary light is 1.25, which is of high consistency with the gray/brown color in the aqueous solutions (bottom panel in Fig. 2c). Under polarized light, the factor can be modulated in a much broader range, from 0.5 to 2.7 (Fig. 2i), giving rise to obvious color changes in the aqueous solutions once aligning the cAuNRs from *y* axis to *z* axis via a magnetic field (Fig. 2c).

### Programmable mechanochromic response to stress

**Fig. 3:**
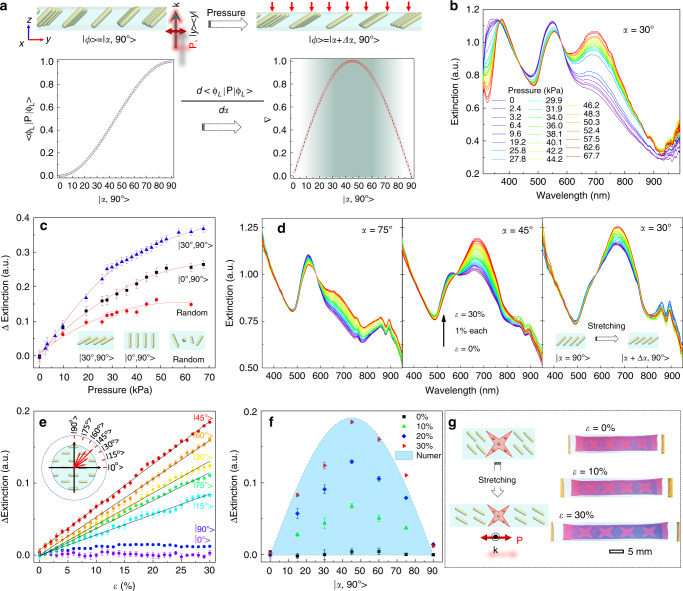
Programming the mechanochromic response by magnetic alignment. **a** The design principles of mechanochromic response of plasmonic films upon pressing and stretching. **b** Extinction spectra of the plasmonic films under different pressures with cAuNRs aligned along 30^o^ to the surface normal. **c** Intensity changes of longitudinal modes when the plasmonic films were subject to different pressures. Insets: cross-section view of the plasmonic films. **d** Extinction spectra of the plasmonic films under different strains with cAuNRs aligned along 75^o^ (left panel), 45^o^ (middle panel), and 30^o^ (right panel) to the stretching direction. Spectra were measured as strain (*ε*) increased from 0% to 30% with a step of 1%. Insets: top view of the plasmonic films during stretching. **e** Summary of intensity changes of cAuNRs under different strains. Arrows in the inset indicate the slopes of the mechanochromic response. **f** Anisotropic mechanochromic response of the plasmonic films enabled by magnetic alignment. The abbreviation, Numer, indicates the numerical results. **g** Orientation-dependent mechanochromic response of plasmonic films enabled by magnetically aligning cAuNRs along pre-designated directions. The top view of the plasmonic film is illustrated in the left panel to show the alignment of cAuNRs. Error bars in **c** and **e** represent the standard deviations from three experimental measurements.

The dependence of plasmonic excitation of cAuNRs on their orientation offers a reliable tool for fabricating mechanochromic devices. To this end, we prepared a cAuNRs/polymer composite film with nanorods aligned along a given direction by UV-curing an aqueous dispersion of cAuNRs and acrylamide under a uniform magnetic field (see Supplementary Discussion IV). The parallel alignment of cAuNRs with the magnetic fields was confirmed by the good agreement between the measured and theoretical extinction of cAuNRs in the films (Supplementary Fig. 13). The SEM images in Supplementary Fig. 14 demonstrated the well-defined orientational order of cAuNRs in the polymer matrices, and further statistical analysis revealed a narrow normal distribution of cAuNRs orientation along the directions of magnetic fields (SD = 2.8^o^). Inspired by the precise alignment of cAuNRs with the applied magnetic fields, we then proposed a simple method to prepare mechanochromic films with optimal and predictable colorimetric responses to stress. As illustrated in the upper panel of Fig. 3a, cAuNRs with a pre-designed orientation (׀*α*, 90^o^ >) will be re-configured to another alignment (׀*α* + Δ*α*, 90^o^ >) with small changes in azimuth angle in response to a unidirectional pressing or stretching. An ideal mechanochromic film is expected to exhibit a substantial change in its optical properties when experiencing a minimal change in its azimuth angle during mechanical perturbation. Therefore, we started by calculating the first derivative of the longitudinal excitation of cAuNRs to optimize the mechanochromic sensitivity. As plotted in Fig. 3a, it approaches maximum and then decreases dramatically as α increases from 0^o^ to 90^o^, suggesting that [30^o^, 60^o^] (slope threshold of 0.9) is the optimal range for engineering mechanochromic film with high sensitivity. To verify this hypothesis, we prepared three plasmonic films, in which cAuNRs were aligned randomly, 0^o^, and 30^o^ to the surface normal. When the films were subjected to various pressures, their plasmonic excitation was monitored in-situ in real-time (Supplementary Fig. 16). The embedded cAuNRs tended to rotate to a horizontal position due to the elastic deformation of the polymer under vertical pressures. In Fig. 3b, as the pressure increased from 0 to 67.7 kPa, the intensity of longitudinal modes of cAuNRs gradually increased. Interestingly, the change of longitudinal excitation (ΔE) under 30^o^ was significantly larger than that of 0^o^ or random orientation (Fig. 3c). This observation is consistent with our theoretical prediction of sensitivity and experimentally verifies the proposed working principle for designing highly sensitive mechanochromic films. We further investigated the mechanochromic response of the films under stretching (see Supplementary Discussion V). The extinction spectra were systematically measured by stretching the film along different directions relative to the rod orientation. Under unidirectional strains (ε), the film elongates along the axial direction and narrows due to the Poisson effect. Therefore, the cAuNRs realign to the axial direction and produce traceable changes in their extinction spectra (Supplementary Fig. 18). In the three representative films in Fig. 3d, the longitudinal and transverse modes were gradually enhanced and suppressed, respectively, when the strain increased to 30%. Figure 3e reveals a linear correlation between ΔE and ε, the fitting slopes of which are highly dependent on the initial alignment (*α*) of cAuNRs. As summarized in the inset of Fig. 3e, the film exhibits anisotropic mechanochromic responses during stretching. More specifically, we observe the maximum slope at 45^o^, and when *α* deviates from this angle, the slope decays to a negligible value (Fig. 3f). The dependence on *α* can be predicted by the first derivative of the longitudinal excitation in Fig. 3a, indicating the general applicability of the mechanochromic film for stress sensing. By utilizing the nonlinear dependence of colorimetric response on *α*, we prepared a film with programmable mechanochromic responses to stress. Differential chromatic responses could be produced by patterning cAuNRs with different orientations within the film. In the specific example shown in Fig. 3g and Supplementary Movie 3, cAuNRs were magnetically aligned vertically (90^o^) in the red stars, and 45^o^ in other parts to the stretching direction. During stretching, the ΔE of cAuNRs in the red stars was negligible while it gradually increased with strains in other areas, resulting in enhanced longitudinal plasmonic excitation and a complementary blue color. Therefore, the plasmonic film exhibited a changing contrast as the strain increased, providing a highly sensitive and vivid colorimetric response.

### Motion-active plasmonic films

**Fig. 4:**
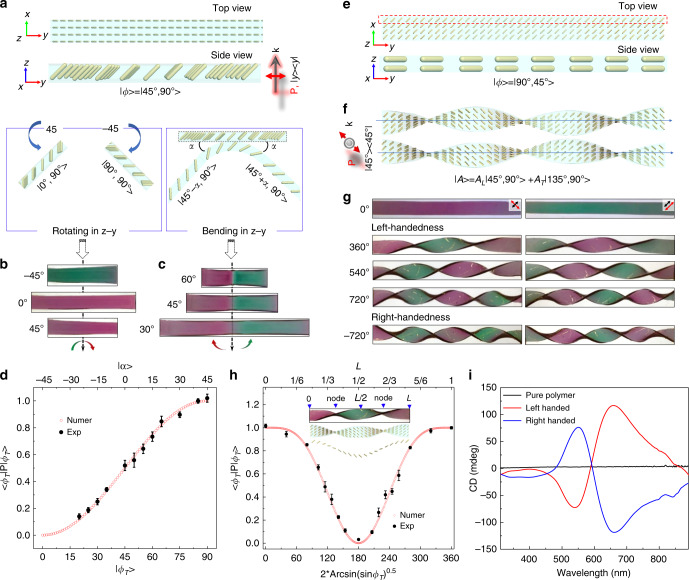
Motion-active plasmonic films. **a** Schematics of the specific arrangement of cAuNRs in the plasmonic film. **b** Top views of the plasmonic films under different rotation angles. **c** Top views of the plasmonic films under different bending angles. **d** The transverse excitation of cAuNRs under different transverse phase angles (*ɸ*_T_). **e** Schematics showing the in-plane 45° arrangement of cAuNRs inside the plasmonic film at the top (top) and side view (bottom). **f** Schematics of left-handed twist (top) and right-handed twist states (bottom). The twisting angle is set at 540°. **g** Digital images of the plasmonic film at initial (top panel), left-handed (middle panel), and right-handed twisting states (bottom panel). The polarization direction and orientation of cAuNRs are illustrated by red and black arrows, correspondingly. **h** Dependence of transverse excitation on localized rotation angle and *y*-coordinates by analyzing the superposition of intensity of transverse and longitudinal resonances to the overall lineshape. Insets: a picture of the twisted plasmonic film and the helical configuration of cAuNRs. **i** CD spectra of pure polymer and plasmonic films under twisted configuration. Error bars in **d** and **h** represent the standard deviations from three experimental measurements.

In addition to simple pressing and stretching, we further demonstrate the versatility of the magnetic alignment approach for preparing mechanochromic devices with programmable colorimetric responses to linear rotation, bending, and nonlinear twisting. Figure 4a illustrates the alignment of cAuNRs along 45^o^ out of plasmonic films, notated as |45^o^, 90^o^ > under *y*-polarization. When the film was rotated by 45^o^, the orientation of cAuNRs became 0^o^. Consequently, the films turned to red as only the transverse mode of cAuNRs was excited (Fig. 4b). At −45^o^, the plasmonic excitation of cAuNRs transited to |90^o^, 90^o^ > . The complementary green color of the longitudinal mode was observed in the films. In contrast to uniform color changes upon rotating, bending induced different colors at the two ends of the film due to the separation of the excitation states. For example, bending the film by 45^o^ downward realigned cAuNRs into vertical,|0^o^, 90^o^ > , and horizontal, |90^o^, 90^o^ > , orientations, which further induced selective transverse (left end) and longitudinal (right end) excitation correspondingly and exerted uniform red and green colors at the two ends (Fig. 4c; Supplementary Movie 4). To confirm the predicted plasmon modes against rotation, we measured the extinction spectra at various rotation angles (Supplementary Fig. 19a, b). As *α* increased, transverse extinction was enhanced while longitudinal excitation was suppressed. The relative extinction was derived by Supplementary Eq. (18) and plotted against the transverse-mode angle (*ɸ*_T_) in Fig. 4d, which further confirms the trigonometric prediction of the bra-ket theorem (Eq. (2)). An excellent agreement was also found between the derived *ɸ*_T_–*α* correlation from Supplementary Eq. (19) and theoretical prediction (Supplementary Fig. 19c), which explicitly demonstrated the linear nature of rotation. Notably, one may expect a similar dependence of the mode angle on the bending or rotation angle because bending essentially induces opposite rotation effects on the two ends of the film (Supplementary Fig. 20).

By comparing rotating and bending, a critical principle became clear: symmetry-breaking along the active axis of mechanical perturbations induces separation of plasmon modes of cAuNRs during that motion (Supplementary Fig. 21). In order to verify this hypothesis, we have constructed a three-dimensional model with cAuNRs aligned 45° within the films (top-view in Fig. 4e). For both left- and right-handed helices formed upon 540° twisting, the aligned cAuNRs were re-configured into a helical form (Fig. 4f). Our further interpretation of the twisting perturbation revealed that the helical configuration was induced by a localized rotation effect. More specifically, twisting the film along its long axis implied localized rotational perturbations with a continuously increased rotating angle (γ), which can be visualized by configuring the three-dimensional orientation of representative rods (highlighted by the red dashed rectangle in Fig. 4e). The rods tend to rotate around the *y* axis by the twisting perturbation (Supplementary Fig. 22a). In this case, whereas the angle between their orientation and *y* axis remained at 45°, *γ* varied as a function of positions, whose dependence can be described as ω*y/L, where *ω* is the twisting angles, *y* is the coordinates of one arbitrary position in the films, and L represents the total length of the film (Supplementary Fig. 22b).

In the experiment, the alignment of cAuNRs was achieved by applying magnetic fields at the designated angle followed by UV fixation. The successful alignment was evidenced by the uniform red and green color across the films under perpendicular and parallel polarization, respectively (Fig. 4g). When the film was twisted, its initial uniform color turned into alternating red and green segments. Due to the symmetric orientation of cAuNRs against the *x*–*y* plane, the same changes of plasmon modes were observed during left-handed and right-handed twisting, thus inducing a handedness-independent mechanochromic response. When the polarizer was rotated by 90° in the *x*–*y* plane, the initial colors of the twisted film turned to the opposite ones. To understand the intrinsic dependence of optical properties on twisting, we measured the space-resolved extinction spectra of twisted films (Supplementary Fig. 23), whose transverse mode was extracted and substituted into Eq. (4). The resulted extinction–local rotation correlation was plotted in Fig. 4h, which followed exactly the profiles predicted by Supplementary Eq. (21). The plasmon resonance of cAuNRs gradually switched from longitudinal mode to transverse mode as the twisting propagated from 0° to 180° inside the helical film. The helical configuration of cAuNRs was evidenced by the appearance of significant circular dichroism (CD) signals in Fig. 4i, and further confirmed by the localized surface electric fields excited at 800 nm and Poynting vectors excited at 630 nm, which represented the strength of longitudinal and transverse resonance, respectively (Supplementary Figs. 24, 25). We examined the quantitative correlation of *γ*–*ɸ*_T_ from two independently measured quantities, the *y*-coordinates of the films and the transverse extinction (Supplementary Fig. 23b, c), and found that such dependence could be well predicted by the theoretical calibration curve calculated from Supplementary Eq. (22) (Supplementary Fig. 23d). During each 180° twisting, one node was formed, which separated two regions with orthogonally aligned cAuNRs as evidenced by the clear color contrast in Supplementary Movie 5. More importantly, twisting exerted nonlinear perturbations to the excitation states of cAuNRs because the dependence of transverse-mode angle (*ɸ*_T_) on local rotation angle (*γ*) was nonlinear.

**Fig. 5:**
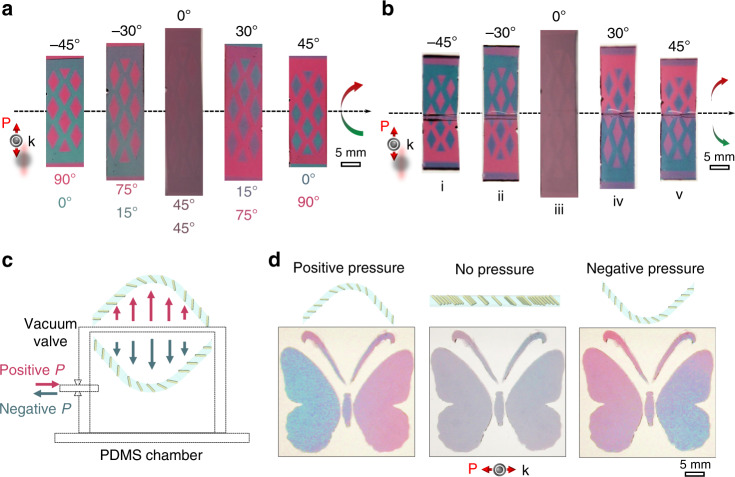
Mechanochromic devices. Top views of the plasmonic films under (**a**) rotation and (**b**) bending. The numbers below the images in (**a**) indicate the longitudinal mode angles (*ɸ*_L_) of cAuNRs embedded in the regions as indicated by the same color of the angles. In (**a**), the plasmonic film was rotated left-handed. In (**b**), negative bending angles (i and ii) indicate bending backward while positive angles (iv and v) indicate bending forward. **c** Scheme showing the mechanochromic pressure sensor. **d** The cAuNRs are aligned 45^o^ to the surface normal in the butterfly patterns (middle panel). When subjected to pressure, the top plasmonic film expands upward or downward and exhibits asymmetric mechanochromic response in the two wings of the butterfly due to the excitation of different plasmon modes of cAuNRs.

More complex colorimetric responses to mechanical motions can be programmed by patterning differently aligned cAuNRs at different locations of the films. As shown in Fig. 5a, the alignment of cAuNRs in the rhombus and background regions was 45° to the top and 45° to the bottom, respectively. Primary gray/brown was observed as both transverse and longitudinal modes were excited in the two regions. When the film was rotated left-hand to 30° and 45°, the rhombus area turned blue, and the background appeared red. Interestingly, the colors switched when the film was rotated to −30° and −45°. The plasmonic excitation of cAuNRs in the two regions diverged from each other as rotation increased, thereby exhibiting the pre-designed images with high contrast. In the case of bending (Fig. 5b), the asymmetric mechanochromic response was observed in the regions separated by the bending axis due to the opposite effect of bending to the plasmonic excitation of cAuNRs with the same orientation in the two regions. Thanks to its solution processability, the fabrication can be easily scaled up to produce centimeter-sized films with programmable mechanochromic responses to bending and rotating (Supplementary Fig. 26 and Supplementary Movie 4).

We further demonstrate the versatility of the system by constructing a mechanochromic film with readable and asymmetric colorimetric responses to pressure change in an air chamber (Fig. 5c). The chamber was made by polysiloxane and glass, with the top opening sealed by a plasmonic film containing cAuNRs uniformly aligned 45° to the surface normal in a “butterfly” pattern. At ambient pressure, the flat film was gray because both transverse and longitudinal modes were excited (Fig. 5d). When air was injected into the chamber, a positive pressure pushed the top layer outward and displayed an asymmetric colorimetric response in the two wings, showing blue on the left and red on the right. In contrast, the color switched in the two wings under negative pressure when air was extracted from the chamber. While this device may find potential use as a simple colorimetric pressure indicator, more complex patterns can be designed based on the convenient magnetic alignment to provide readable colorimetric responses that allow qualitative estimation of the applied pressure (Supplementary Fig. 27, Supplementary Movie 7).

## Discussion

In this work, we report a direct colloidal synthesis method to prepare hybrid magnetic-plasmonic nanostructures with well-defined morphologies and physical properties. On the basis of the unique properties of the hybrid nanorods, we have further proposed a reliable method to prepare mechanochromic films with controllable colorimetric responses towards linear and nonlinear mechanical motions and deformations. The plasmonic excitation of Au nanorods can be conveniently tuned by a magnetic field, producing selective excitation of plasmon modes in both the colloidal dispersions and polymer matrices. This synthetic method combines the advantages of conventional confined growth with the flexibility of structural engineering of nanomaterials, which is expected to produce a number of hybrid nanostructures by simply changing the initial templates and chemical components of the secondary metals. The enhanced colloidal stability of hybrid magnetic-plasmonic nanorods enables us to magnetically align them along pre-designed directions within polymer films. The asymmetric alignment of anisotropic plasmonic nanostructures about the active axis of external mechanical stimuli induces the excitation of different resonance modes, producing readable color changes. The incorporation of hybrid nanostructures and their magnetic alignment are compatible with the current fabrication processes of soft actuators, robots, and biomimetic systems. In addition, the contactless, fast, and reversible magnetic interactions allow colloidal nanoparticles to be efficiently aligned and patterned in polymer matrices, making them potentially useful for various applications, such as displays, sensors and actuators, anti-counterfeiting devices, and biomimetic systems with simultaneous shape and color changes.

## Methods

### Synthesis of FeOOH nanorods

In total, 10.8 g of FeCl_3_·6H_2_O was dissolved in 400 mL of deionized water and heated to 87 °C in an oven for 18 h. After that, FeOOH precipitated at the bottom, and the supernatant was discarded. The precipitation was washed in deionized water three times at 11,000 rpm for 15 min and finally dispersed in 40 mL of DI water.

### Silica coating on FeOOH nanorods and reduction

To form uniform SiO_2_ coating, FeOOH was modified by PAA first. Typically, 216 mg of PAA (~1800) was dissolved in 600 mL of DI water, and 10 mL of FeOOH aqueous dispersion was added afterward. The solution was magnetically stirred overnight. FeOOH was recovered by centrifugation and washed with DI water three times at 11,000 rpm for 15 min The PAA-modified FeOOH was dispersed in 12 mL of DI water. For silica coating, 4 mL of FeOOH dispersion was concentrated into 2 mL and added to 40 ml of ethanol followed by 250 µL ammonium solution (28%). In all, 125 µL TEOS was added for 4-nm SiO_2_ coating or 250 µL of TEOS was added twice with a 1-h interval for 8-nm SiO_2_ coating. To achieve 12-nm SiO_2_ coating, 2 mL of FeOOH dispersion was added in a 20 mL of ethanol followed by 250 µL of ammonium solution and 150 µL of TEOS twice with a 1-h interval. The mixture was magnetically stirred overnight. Afterward, FeOOH@SiO_2_ was centrifugated out at 14,500 rpm for 10 min and washed with ethanol once and water three times. FeOOH@SiO_2_ was reduced to magnetic nanorods in DEG at 220 °C. In total, 15 mL of DEG was heated to 220 °C under nitrogen, to which 250 µL of FeOOH@SiO_2_ aqueous solution was injected. The reduction was kept for 6 h under nitrogen protection. The final product was washed by ethanol and water three times and dispersed in 12 mL of ethanol.

### APTES modification

Typically, the dispersion of magnetic nanorods in ethanol was added into 50 mL of ethanol. Then it was heated to 80 °C and 200 µL of APTES was added quickly. The surface modification usually took 5 h under nitrogen protection. Afterward, the product was washed by ethanol four times and dispersed in 12 mL of ethanol. The protonation of the amino group rendered the nanorods positively charges, which were capable of attracting the negatively charged Au seeds through electrostatic interaction.

### Au seed preparation

The Au seeds were prepared according to a previously reported method^42^. To 45 mL of Milli-Q water, 12 µL of THPC and 250 µL of NaOH (2 M) were added. After 5 min, 2 mL HAuCl_4_ (1%) was added. The solution was covered by foil and stirred overnight. Afterward, it was stored at 4 °C as stock seed solution.

### Au seed attachment and PVP modification

In all, 3 mL of magnetic nanorods in ethanol was centrifugated and washed with DI water twice. It was dispersed in 5 mL of DI water and added into 10 mL of Au seed stock solution. The mixture was stirred about 1 h. Negative-charged Au seeds were attached to the surface of magnetic nanorods through electrostatic interaction. Excess Au seeds were discarded after centrifugation at 145 rpm for 10 min Afterward, 5 mL of DI water was added to disperse the solids. It was then transferred to 10 mL of PVP solution (20 mg mL^−1^, MW = 10,000) under sonication. The mixture was stirred overnight at room temperature.

### RF coating

Excess PVP was removed by centrifuge at 14,500 rpm for 10 min. The solids were washed by DI water twice and finally dispersed in 28 mL of DI water. In total, 13 mg of R and 18 µL of F were added into that dispersion sequentially. After that, the mixture was heated to 50 °C, and then 100 µL ammonium solution (2.8%) was added quickly. The reactive was kept at 50 °C for 2 h and heated to 100 °C. The condensation at 100 °C took 5 h. Meanwhile, the SiO_2_ shell was etched completely, forming a gap between magnetic nanorods and RF shell with Au seeds inside due to the week alkaline condition. The final product was washed by Milli-Q water three times and dispersed in 2 mL Milli-Q water as seed solution.

### Confined growth of cAuNRs

The seeded growth was carried out based on work reported previously^44^. Typically for growth of concave AuRNs to the full length, chemicals were added into 2 mL of Milli Q water in the following sequence, 500 µL of PVP (20 mg mL^−1^, MW = 10,000), 33 µL of KI (0.2 M), 33 µL of AA (0.1 M), 5 µL of HAuCl_4_(0.25 M), and finally 25 µL of seed solution. The growth usually took ~5 min. If decreasing the amount of AA, KI, and HAuCl_4_ proportionally, concave AuRNs with different aspect ratio were achieved (Fig. 1g, h; Supplementary Fig. 5).

### Preparation of plasmonic films

To prepare the plasmonic films, 250 mg of AM, 14 mg of BIS, and 3 µL of 2-Hydroxy-2-methylpropiophenone were dissolved in 1 mL of DEG. While AM is the monomer of the polymer, BIS and 2-hydroxy-2-methylpropiophenone act as a cross-linking agent and photoinitiator (PI), respectively. In all, 1 mL of cAuNRs colloidal dispersion was centrifugated at 9000 rpm for 3 min, and supernatant was removed. Then, 200 µL of the precursor solution was added and sonicated for ~ 15 s to fully disperse the colloidal nanoparticles. To prepare the solid films, the solution containing magnetic–plasmonic nanorods was sandwiched between glass slides with a spacer (~1 mm), which was exposed to UV light (254 nm) for 1 min. For lithography, photomask was first placed above the cover glass before UV irradiation. After the first exposure, the mask was removed, and different magnetic fields with pre-designed directions were applied, followed by another UV exposure. The sequential magnetic alignment, UV exposure could be programmed to control the alignment of magnetic–plasmonic nanorods in specific locations. Experimentally, magnetic alignment was achieved by placing the precursor solution into the center of two identical permanent magnets to ensure the uniform alignment in a parallel fashion. The field strength was measured to be 25 mT (250 G). Before UV irradiation, magnetic alignment was balanced for ~ 10 s, and magnetic fields were not removed during polymerization. To prepare the mechanochromic devises, PDMS films were used. In a typical process, silicone elastomer curing agent and silicone elastomer base were thoroughly mixed with a mass ratio of 1:10. The mixture was cured at 60 °C for 2 h^47^.

### Bra-ket notation of plasmonic excitation

The linear polarization effect and the selective excitation of plasmon modes shown in this work can be described by bra-ket notation in mathematics. Referring to Supplementary Fig. 4, the light is incident along the *y* axis and polarized along the *z* axis. The linear polarizer can be denoted as

5 $$P = |z > < z|,$$

where *z* is the polarization direction. In the experiment, the cAuNRs were rotated in *x*–*y* and *y*–*z* plane by magnetic control. Specifically, in the spherical coordinate, the angle between *z* axis and the long axis of nanorods is defined as *α*, while it is defined as *Ɵ* for the angle between the *x* axis and the long axis of projection of Au nanorods in the *x*–*y* plane. In this scenario, the state of longitudinal and transverse mode of plasmon resonance shall be denoted as |α, *Ɵ* > , |90^o^ + *α*, *Ɵ* > . The state of plasmon resonance of concave AuNRs under arbitrary orientation is simply the sum of individual mode multiplied by a magnitude term, *A*_L_ for longitudinal mode and *A*_T_ for transverse mode. Each term can be expressed as a linear combination of three orthogonal eigenvectors, ׀*x* > , ׀*y* > and ׀*z* > . Therefore, for nanorods, the state function of plasmonic extinction, simply the sum of state function of individual plasmon resonance mode (transverse and longitudinal), can be denoted as

6 $$|\varPhi > = |\varPhi _{\mathrm{L}} > + |\varPhi _{\mathrm{T}} > = {A}_{\mathrm{L}}|{\alpha},{\theta} > + {A}_{\mathrm{T}}|90^o + \alpha ,\theta > $$

As discussed above, ׀α, *Ɵ* > = sin*α*cos*Ɵ*׀*x* > + sin*α*sin*Ɵ*׀*y* > + cos*α*׀*z* > . The kets, ׀*x* > , ׀*y* > and ׀*z* > , satisfy orthonormality condition. *A*_L_ and *A*_T_ are the maximum extinction coefficient of longitudinal and transverse excitation, respectively. Since the longitudinal excitation of hybrid nanorods is parallel to the long axis, its orientation state is the same as that of the rods. In the case of transverse modes, however, an angle of 90^o^ is added to *α* as transverse modes are perpendicular to the orientation of nanorods. Accordingly, their maximum extinction intensity in the spectra can be described as *A*_L_^2^ and *A*_T_^2^.

When *z*-polarized light passes through the solution to be measured, the longitudinal and transverse mode will be excited and features several absorption peaks in the UV–Vis spectra. These processes can be mathematically interpreted as the polarizer operator, *P*, acting on the state of nanorods, |$\varPhi$>, and the generated new ket defines the excited state of plasmon resonance. The total absorbance is derived as:

7 $$\left| A {> } \right. =	\, \left.P \right|\varPhi _{\mathrm{L}} > + P|\varPhi _{\mathrm{T}} > \\ =	\, |z > < z|({A}_{\mathrm{L}}|{\alpha},{\theta} > + {A}_{\mathrm{T}}|90^o + \alpha ,\theta > ) \\ =	 \, \left| {z > < z} \right|\left( {A}_{\mathrm{L}}{\mathrm{sin}}\alpha {\mathrm{cos}}\theta |x > + {A}_{\mathrm{L}}{\mathrm{sin}}\alpha {\mathrm{sin}}\theta \left| {y > + {A}_{\mathrm{L}}{\mathrm{cos}}\alpha } \right|z > \right. \\ 	+ \left. {A}_{\mathrm{T}}{\mathrm{sin}}(\alpha + 90^o){\mathrm{cos}}\theta |x > + {A}_{\mathrm{T}}{\mathrm{sin}}(\alpha + 90^o){\mathrm{sin}}\theta |y > + {A}_{\mathrm{T}}{\mathrm{cos}}(\alpha + 90^o)|z > \right) \\ =	\, {A}_{\mathrm{L}}{\mathrm{sin}}\alpha {\mathrm{cos}}\theta \left| {z > < z} \right|x > + {A}_{\mathrm{L}}{\mathrm{sin}}\alpha {\mathrm{sin}}\theta \left| {z > < z\left| {y > + {A}_{\mathrm{L}}{\mathrm{cos}}\alpha } \right|z > < z} \right|z > \\ 	+ {A}_{\mathrm{T}}{\mathrm{cos}}\alpha {\mathrm{cos}}\theta \left| {z > < z} \right|x > + {A}_{\mathrm{T}}{\mathrm{cos}}\alpha {\mathrm{sin}}\theta \left| {z > < z} \right|y > - {A}_{\mathrm{T}}{\mathrm{sin}}\alpha \left| {z > < z} \right|z > \\ =	\, {A}_{\mathrm{L}}{\mathrm{cos}}\alpha |z > - {A}_{\mathrm{T}}{\mathrm{sin}}\alpha |z> $$

As seen here, the absorbance is characterized by two terms with the same phase but different coefficients. Whereas the first one defines the longitudinal mode, the second indicates the transverse one. The physical meaning of ket, ׀*z* > , is that the excitation of plasmon resonance is along the *z* axis for both modes. *A*_L_cosα and *A*_T_sinα are absorption efficiency for the longitudinal and transverse modes. If *α* is 0, which means the AuNRs are parallel to the *z* axis, the second term will be zero. The only longitudinal mode is excited, and the absorption efficiency reaches maximum, *A*_L_, indicating the highest intensity for the longitudinal mode.

### Deriving expectation value of excitation

Bearing this in mind, we show, in the following, how the bra-ket notation helps to describe the selective excitation of plasmon resonance of nanostructures under linearly polarized light. The expectation value of the polarization operator, **P**, for an orientational state, $\varPhi$, of cAuNRs is the sum of two terms, transverse and longitudinal modes and can be described mathematically as:

8 $$< A(\alpha ,\theta ) > =	 \, < {A}_{\mathrm{L}}(\alpha ,\theta ) > + < {A}_{\mathrm{T}}(\alpha ,\theta ) > \\ =	 \, < \varPhi _L\left| {\mathrm{P}} \right|\varPhi _{\mathrm{L}} > + < \varPhi _{\mathrm{T}}\left| {\mathrm{P}} \right|\varPhi _{\mathrm{T}} > \\ =	 \, < \varPhi _{\mathrm{L}}|{A}_{\mathrm{L}}{\mathrm{cos}}\alpha |z > + < \varPhi _{\mathrm{T}}| - {A}_{\mathrm{T}}{\mathrm{sin}}\alpha |z > \\ =	 \, \left( {{A}_{\mathrm{L}}{\mathrm{sin}}\alpha {\mathrm{cos}}\theta < x{\mathrm{|}} + {A}_{\mathrm{L}}{\mathrm{sin}}\alpha {\mathrm{sin}}\theta < y| + {A}_{\mathrm{L}}{\mathrm{cos}}\alpha < z|} \right){A}_{\mathrm{L}}{\mathrm{cos}}\alpha |z > \\ 	 + \left( {{A}_{\mathrm{T}}{\mathrm{cos}}\alpha {\mathrm{sin}}\theta < x} \right. \left. { \, + \, {A}_{\mathrm{T}}{\mathrm{cos}}\alpha {\mathrm{cos}}\theta < y| - {A}_{\mathrm{T}}{\mathrm{sin}}\alpha < z|} \right)\left( { - {A}_{\mathrm{T}}{\mathrm{sin}}\alpha |z > } \right)\\ =	 \, {A}^2_{\mathrm{L}}{{\mathrm{cos}}^2\alpha + {A}_{\mathrm{T}}^2}{\mathrm{sin}}^2\alpha$$

The expectation value of absorption, or experimental measurable, is solely dependent on the azimuthal angle, *α*. *I*_L_ and* I*_T_ represent the maximum intensity of longitudinal and transverse modes. For *α* *ϵ* [0^o^, 90^o^], the expectation value of plasmonic excitation of either transverse or longitudinal mode intrinsically follows:

9 $$< A\left( {\alpha ,\theta } \right) > + < A\left( {90^o - \alpha ,\theta } \right) > = 2 < A(45^o,\theta ) > $$

This derived symmetry relation helps to deduce the expectation of plasmonic excitations.

### Simulation of excited states under different orientation angles

The simulated spectra of cAuNRs under various orientations are computed by the finite-element frequency-domain method (Comsol Multiphysics). To explicitly model component diversity and the structural complexity, morphologies and size are all derived from TEM images. The shape of initial AuNRs is derived as a flat rod terminated with two half ellipsoids (Supplementary Fig. 5) because the morphology of cAuNRs observed from TEM images is neither perfect rod nor an ideal ellipsoid. Basically, the two ellipsoids on the ends can refine the sharpness and make the model more feasible. To create the concavity in the model, we removed another cutting rod with a radius of 10 nm was built atop the AuNRs, and the overlay of the two. As shown in Supplementary Fig. 5, the sharp edge around the concave was further rounded by another rod with a radius of 50 nm to mimic the smooth surface of cAuNRs as evidenced by TEM images in Supplementary Fig. 2d. Magnetic nanorod was modeled as a cylinder with a large aspect ratio and two spherical ends. The overall size is 110 nm in length and 20 nm in width.

The material domains were defined separately to endow them basic physical properties, like relative permittivity or refractive index. The interpolation functions for the complex refractive index of gold is taken from the Optical Materials Database. The refractive index of the surrounding is set as 1.5 to mimic the presence of the polymer shell. A plane electromagnetic wave propagates along the *x* axis, and is polarized in the *z* axis. The wavelength of the electromagnetic wave was swept from 400 nm to 1000 nm with a 10-nm step. The absorption cross-section was computed by integrating power loss density over the volume of Au and Fe_3_O_4_ nanorods. Scattering cross-section was defined as integral of the dot product of surface normal vector and Poynting vector over the close surface of both Au and Fe_3_O_4_ nanorods. The extinction cross-section is simply the sum of the two. After computing the scattering field by solving Maxwell’s equations, the electric field norm and Poynting vector at a specific wavelength were plotted to visualize the localized surface plasmon resonance and scattering around the concave structure. The extinction, absorption, and scattering cross-section were plotted in Supplementary Fig. 9a–c, respectively.

## Supplementary information

Supplementary Information

Peer Review File

Description of Additional Supplementary Files

Supplementary Movie 1

Supplementary Movie 2

Supplementary Movie 3

Supplementary Movie 4

Supplementary Movie 5

Supplementary Movie 6

Supplementary Movie 7

## Data Availability

All data generated and analyzed during this study are included in this published article (and its Supplementary Information files) and are available from the corresponding author on reasonable request.
