# Supplementary material for: Coupling magnetic and plasmonic anisotropy in hybrid nanorods for mechanochromic responses
Source: Nat Commun. 2020 Jun 8;11:2883. doi: 10.1038/s41467-020-16678-8 (PMC7280256; doi:10.1038/s41467-020-16678-8)
Supplement: Supplementary file 1 — Supplementary Information [file 41467_2020_16678_MOESM1_ESM.pdf]

Supplementary Information

Coupling magnetic and plasmonic anisotropy in hybrid nanorods for mechanochromic responses

Li et al.

Supplementary Methods

I. Synthesis and characterization of magnetic-plasmonic nanostructures S4

II. Bra-ket notation of plasmonic excitation under linear polarized light S6

Supplementary Discussion

I. Understanding the plasmonic properties of cAuNRs S9

II. Coupling magnetic and plasmonic anisotropy within nanorods S10

III. Quantifying the correlation between excited states and orientation angles S11

IV. Fixing the orientation of cAuNRs by photolithography S13

V. Processing of time- and space-resolved spectra under mechanical perturbation S15

VI. Fabricating programmable mechanochromic devices S19

Supplementary Figures

Supplementary Figure 1 Characterization of Fe_3O_4@SiO_2 magnetic nanorods S22

Supplementary Figure 2 TEM and UV-Vis spectra of cAuNRs S23

Supplementary Figure 3 Microscopic images, DLS and zeta potential of cAuNRs S24

Supplementary Figure 4 Bra-ket notation of linear polarization S25

Supplementary Figure 5 Modeling of cAuNRs S26

Supplementary Figure 6 Deriving the expectation value of plasmonic excitation based on simulation S27

Supplementary Figure 7 Simulated ECS, ACS and SCS of cAuNRs S28

Supplementary Figure 8 Magnetic and plasmonic anisotropy of hybrid nanorods S29

Supplementary Figure 9 Simulation of orientation-dependent plasmonic excitation of cAuNRs S30

Supplementary Figure 10 Orientation-dependent plasmonic excitation of cAuNRs S31

Supplementary Figure 11 Orientation-dependent plasmonic excitation of cAuNRs S32

Supplementary Figure 12 Tunability factor of cAuNRs S33

Supplementary Figure 13 Plasmonic excitation of plasmonic films S34

Supplementary Figure 14 SEM images of polymer films and plasmonic films S37

Supplementary Figure 15 Polarization-dependent coloration of plasmonic films S38

Supplementary Figure 16 Setup for measuring mechanochromic response to pressure and strain S39

Supplementary Figure 17 Extinction spectra of the plasmonic films under different pressures S40

Supplementary Figure 18 Extinction spectra of the plasmonic films under different strains S41

Supplementary Figure 19 Spectral analysis of plasmonic film upon rotating perturbation S42

Supplementary Figure 20 Separation of excited states of plasmonic film upon bending S43

Supplementary Figure 21 Symmetry-breaking for unique mechanochromic response S44

Supplementary Figure 22 Angle analysis in twisted plasmonic films S45

Supplementary Figure 23 Analyzing nonlinear twisting perturbation S46

Supplementary Figure 24 CD spectra of twisted films S47

Supplementary Figure 25 Electric filed distribution and Poynting vectors of twisted cAuNRs S48

Supplementary Figure 26 Programmable mechanochromic response enabled by magnetic alignment S49

Supplementary Figure 27 Mechanochromic devices S50

Supplementary Methods

I. Synthesis and characterization of magnetic-plasmonic nanostructures

Chemicals: Iron chloride hexahydrate ($FeCl_3 \cdot 6H_2O$), polyacrylic acid (PAA, MW=1800), tetraethyl orthosilicate ($C_8H_{20}O_4Si$, TEOS), 3-aminopropyl-triethoxysilane ($C_9H_{23}NO_3Si$, APTES), sodium hydroxide (NaOH), polyvinylpyrrolidone (PVP, MW=10000), resorcinol (R), formaldehyde (F), L-Ascorbic acid (AA), 2-Hydroxy-2-methylpropiophenone and Tetrakis(hydroxymethyl)phosphonium chloride (THPC) were purchased from Sigma-Aldrich. Ammonium hydroxide ($NH_3 \cdot H_2O$) and potassium iodide (KI) were purchased from Fisher Scientific. Chloroauric (III) acid trihydrate ($HAuCl_4 \cdot 3H_2O$), ethylene glycol (EG) and diethylene glycol (DEG) was from Acros Organics. Acrylamide (AM) and N,N'-Methylenebisacrylamide (BIS) were purchased from Fluka. Ethanol was purchased from Decon Labs. SYLGARD 184 silicone elastomer curing agent and SYLGARD 184 silicone elastomer base were purchased from dow silicones corporation. All chemicals are used directly without further purification.

Preparation of slices of plasmonic films: To characterize the alignment of cAuNRs inside the plasmonic films, we prepared slices of the as-made plasmonic films of cuboid shape by cutting the films along the width. Pure polymer film and film with random cAuNRs were prepared for control experiment. Another two polymer films were prepared by applying perpendicular and parallel magnetic fields to the length of the as-made films. The slices were characterized under SEM. Since they were cut all along the width, we expected to observe two different orientation of cAuNRs inside these two films.

Characterization: TEM images were taken on Tecnai 12 transmission electron microscope operating at 120 kV. Ultraviolet-visible-near infrared (UV-Vis-NIR) spectra were measured by Ocean Optics HR2000 CG-UV-NIR high-resolution spectrometer. The same instrument was used for in-situ spectra to monitor the growth dynamics. All spectra were taken on an aqueous dispersion of nanoparticles. For measuring the spectra under polarized light, a commercial polarizer was introduced right in front of the sample. Meanwhile, a magnet was applied to the aqueous suspension at about 1 cm (~ 25 mT). Optical microscopic images were acquired by ZEISS microscopy. Circular dichroism (CD) spectra were measured using Jasco J-815 CD spectrophotometer. SEM images was taken on scanning electron microscopy NovaNanoSEM 450 operating at 10 kV. Dynamic light scattering (DLS) and zeta potential measurement were

conducted in Delsa NanoC Particle Analyzer from Beckman Coulter. The magnetization curve was measured at room temperature (RT) on a squid magnetometer and zero-field-cooling and field-cooling (ZFC/FC) curve was measured at 75 Oe from 10 K to 300 K.

Magnetic anisotropy of Fe_3O_4@SiO_2 nanorods: The TEM image and XRD pattern of Fe_3O_4@SiO_2 was shown in **Supplementary Fig. 1b** and **1c**. The porous structures and broad, weak diffraction peaks suggest that Fe_3O_4@SiO_2 may have a poor crystalline. Accordingly to our previous work, the Fe_3O_4 magetic nanorods were polycrystalline with a crystal domain size of ~6.8 nm[3]. To characterize the magnetic properties of Fe_3O_4@SiO_2 nanorods, we measured the magnetization curve at room temperature (**Supplementary Fig. 1d**). Their saturation magnetization was 16 emu g^{-1}. The low saturated magnetization may be due to the polycrystalline of Fe_3O_4 cores and the presence of SiO_2 shells, which cound lower the overall magnetization by providing more mass without any magnetization contribution. In addition, we further measured ZFC/FC curve of the Fe_3O_4@SiO_2 nanorods at 75 Oe from 10 K to 300 K. As shown in **Supplementary Fig. 1e**, the broad ZFC curve and the splitting of ZFC/FC curve indicate the large variation in the size of crystal domains, which induces a broad distribution of blocking temperature (T_B). The blocking temperature is defined as the temperature when the ZFC curve approached the maximum. In the case of sufficiently small nanolarticles or nanoparticles with sufficiently small magnetic domains, the magnetization will thermally fluctate. The time between two random flip directions is defined as Néel relaxation time (τ_N). When the ZFC curve approaches the maximum, the measurement time (τ_M) is equal to τ_N, which determines the transition from the blocked state to the superparamagnetism state. At a given temperature that is lower than T_B, it is under blocked state. Under the temperature above the T_B, it is under superparamagnetism state. As shown in **Supplementary Fig. 1e**, the measured T_B is lower than room teperature (295 K), indicating that Fe_3O_4@SiO_2 nanorods are superparamagnetic. Based on the classical formula of Néel–Arrhenius equation, $E_B = T_B k_B \ln(t_0/\tau_0)$[4], we can estimate the magnetic anisotropy energy if the interparticle interactions and the polycrystalline character of Fe_3O_4@SiO_2 nanorods were not taken into consideration. In this equation, k_B is the Boltzmann constant (1.38064852×10^{-23} J K^{-1}), t_0 the time scale of measurement (2 s during measurement) and τ_0 the microscopic jump time (10^{-9} to 10^{-11} s). The magnetic anisotropy energy is calculated to be 6.22×10^{-20} J when $\tau_0 = 10^{-10}$ s and measured T_B is 190 K. Furthermore, the magnetic anisotropy constant (K) is calculated to be 1.8 kJ m^{-3} by the

equation, E_B=KV, where V is the volume of nanorods, considering the Fe_3O_4@SiO_2 nanorods as ideal cylinder with radius of 10 nm and height of 110 nm. If the interparticle interaction is taken into account, the Vogel-Fulcher law is better to describe the magnetic anisotropy energy: $E_B=(T_B-T_0)k_B\ln(t_0/\tau_0)$, where T_0 is introduced to calibrate the effect of interparticle interaction[5]. Under this scenario, the true magnetic anisotropy energy and the magnetic anisotropy constant should be smaller than the values estimeted by Néel–Arrhenius equation. Even under Néel–Arrhenius equation model, however, the calculated magnetic anisotropy constant is one order smaller than the theoretical magnetocrystalline anistropy constant (11 kJ m^{-3}) of Fe_3O_4[6-8]. Therefore, it is resonable to conclude that the shape anisotropy dominates in the magnetic anisotropy properties of Fe_3O_4@SiO_2 nanorods and the magnetocrystalline anisotropy is not significant considering their poor crystallinity.

II. Bra-ket notation of plasmonic excitation under linear polarized light

Notation of linear polarization of light: The bra-ket notation is used to describe the linear polarization effect of light. In the 3D Cartesian coordinates, the state of orientation (denoted as |ϕ>) of hybrid nanorods and their plasmonic excitation (denoted as |A>), including both longitudinal ($|A_L>$) and transverse modes ($|A_T>$), can be fully described by a complete set of basis kets, |x>, |y> and |z>. Physically, each basis function indicates one of the three unit column vectors in 3D space. Therefore, they can also be defined in the matrix notation as:

$$|x>= \begin{matrix}1\\0\\0\end{matrix}; |y>= \begin{matrix}0\\1\\0\end{matrix}\ and\ |z>= \begin{matrix}0\\0\\1\end{matrix} \qquad (1)$$

The bra is the conjugate of the ket with the sample symbol and can be expressed as row vectors:

<x|=(1,0,0); <y|= (0,1,0) and <z|=(0,0,1)

Basically, an arbitrary state of orientation of hybrid nanorods and their plasmonic excitation can be expressed by the linear combination of the three unit column vectors. There is, apparently, a fundamental property associated with the three basis functions. They satisfy the orthonormality condition:

$$<n|m>=\delta_{nm};\ \delta_{nm}=1 \text{ when } n=m \text{ and } 0 \text{ when } n\neq m, \qquad (2)$$

where the kets, |n> and |m>, denote one of the three basis functions, |x>, |y> and |z> in the 3D Cartesian coordinates. The expression, <n|m>, is the dot product (also called scale product) of the corresponding bra and ket. In this expression, δ_{nm} is the Kronecker delta. And, the state function of an arbitrary orientation can be denoted as |ϕ>=|α,Θ> (**Fig. 2a**), which can be further expanded as linear combination of the three basis functions by using the correlation between 3D Cartesian coordinates and polar coordinates:

$$|\alpha, \Theta >= sin\alpha cos\Theta|x > +sin\alpha sin\Theta|y > +cos\alpha|z > \quad (3)$$

Similarly, a linear polarizer in the x-y plane with an angle, Θ, to the x axis can be described as an operator, P_Θ=|Θ><Θ| and the state function, |Θ>, can be further described as |Θ>=cosΘ|x>+sinΘ|y>. Therefore, the linear polarizer operator, P_Θ, can be expanded as:

$$P_\Theta = |\Theta >< \Theta|$$

$$= (cos\Theta|x > +\sin\Theta|y >)(\cos\Theta < x| + \sin\Theta < y|)$$

$$= cos^2\Theta|x >< x| + \cos\Theta\sin\Theta|x >< y| + \sin\Theta\cos\Theta|y >< x| + sin^2\Theta|y >< y| \quad (4)$$

With the definition of state functions and the linear polarizer operator, we can easily derive the final states of a polarized right passing through a sequence of polarizers. For example, when Θ=0°, P_0= P_x=|x><x|. When, Θ=90°, P_{90}= P_y=|y><y|. For a x-polarized light incident along z-axis, the final state of the light after successively passing through P_x, P_{45^o} and P_y can be derived as:

$$|\psi > = P_y P_{45^o} P_x |x >$$

$$= P_y P_{45^o}(|x >< x|x >)$$

$$= P_y P_{45^o}|x >$$

$$= P_y(cos^2 45^o|x >< x| + \cos 45^o \sin 45^o|x >< y| + \sin 45^o \cos 45^o|y >< x| + sin^2 45^o|y >< y|)|x >$$

$$= P_y\left(\frac{1}{2}|x > + \frac{1}{2}|y >\right)$$

$$= |y >< y|(\frac{1}{2}|x > + \frac{1}{2}|y >)$$

$$= \frac{1}{2} |y > \quad (5)$$

In the result, 1/2 indicates the probability coefficient and its square value is the intensity or the probability of light passing through the polarizers. The final polarization is determined by the ket, |y>, which predicts a y-polarized light.

Deriving expectation value of transverse excitation under random dispersion: The striking feature of confined seeded growth inside RF polymer shell with magnetic nanorods as a steric impediment is the longitudinal concave structure of AuNRs, which consequently creates another unconventional transverse mode. Those two transverse modes are not resolvable yet considering the intrinsic rotational freedom of nanorods. In the experiment, what has been measured is the average value of absorption of all the different rotational configurations. The expectation value of absorption can be solved by the integral over the whole angular space and given as

$< \Omega >= \frac{\int_0^{2\pi} A_\alpha d\alpha}{\int_0^{2\pi} d\alpha}$, where A_α means the absorbance of nanorods under a rotational angle, α. (6)

To simulate the measured absorption of two transverse modes, we choose seven representative angles in the first quadrant. The absorbance of each configuration is calculated by only exciting the transverse modes. More specifically, the configuration of rotation state follows random dispersion. In other words, the popularity or probability of certain configuration is equal. The complex integral over whole angular space reduces to a simple sum of several states in the first quadrant once taking the symmetry of fours quadrants into account. In this case, if only seven states are considered, the state function of AuNRs can be written as

$$|\phi >= \sum_{i=0}^{7} \frac{1}{\sqrt{7}} |\alpha_i > , \quad (7)$$

where $\frac{1}{\sqrt{7}}$ means dispersion coefficient, whose square determines the popularity or probability. Simply, the absorption of transverse modes of seven chosen rotation configurations are simulated individually and averaged to represent the transverse mode of measurement. In the case of random dispersion of cAuNRs in the suspension, the orientational state function is not an eigenfunction of polarization operator anymore. Under this scenario, however, it can be expressed as a linear combination of eigenfunctions of the operator, *P*, with the dispersion coefficient and the expectation value is simply the weighted sum of the eigenvalues of *P*, the

contribution of a particular orientational state to the sun being determined by the square modulus of dispersion coefficient. Considering the symmetry relation discussed above, the sum over the states simply reduces to a single value as

$$< \Omega >= \frac{1}{7}\sum_{\alpha=0}^{90^o} < A(\alpha,\theta) >=< A(45^o,\theta) > \quad (8)$$

That is the reason why the overall lineshape of the simulated extinction spectra is identical to extinction lineshape of cAuNRs under 45° orientation in **Supplementary Fig. 6**.

Supplementary Discussion

I. Understanding the plasmonic properties of cAuNRs

One interesting feature of cAuNRs is the appearance of the new peak at ~630 nm. To analyze this unconventional plasmon mode, we calculated the extinction cross section (ECS) of the transverse modes of cAuNRs under different orientations by finite-element method (**Supplementary Fig**. **5** for the 3D models). As shown in **Supplementary Fig**. **6 and 7**, the angle between y-axis and line connecting the centers of magnetic and Au nanorods was highlighted to define the transverse excitation. At 90°, only classic transverse excitation was observed at 525 nm in the simulated spectra (**Supplementary Fig**. **7a**). At 0°, however, the new peak with the strongest intensity emerged at 630 nm. Although these two transverse plasmon modes have similar resonance intensity in the measured spectra, two different optical processes are recognized by further calculating their scattering (SCS) and absorption (ACS) cross section. As shown in **Supplementary Fig**. **7b**, the intensity of the SCS of cAuNRs at 630 nm is highly dependent on the orientation angle with a much stronger average signal compared to that at 525 nm, implying a scattering-dominated plasmonic extinction at the second transverse mode. However, the first transverse mode mainly results from absorption as evidenced by the higher average ACS intensity in **Supplementary Fig**. **7c**. The unique concave structures modified the classic transverse resonance slightly but surprisingly created another mode at the longer wavelength with contribution mainly from scattering. Such scattering is significantly localized in the concave surface as directly visualized by the 3D mapping of Poynting vectors (S, also described as energy flux vectors) in **Supplementary Fig**. **7d**. It is interesting to point out that under the configuration of |0°>, the power flow is strong around the concave surface and

ignorable around the other parts. As the angle increases to 90°, the magnitude decreases dramatically, which is consistent with the simulated SCS in **Supplementary Fig**. **7b**. Notably, similar additional transverse modes were also reported in AuNRs with concave structures synthesized by chemical methods[9, 10].

II. Coupling magnetic and plasmonic anisotropy within nanorods

For magnetic nanoparticles exposed to an external magnetic field (H), the induced magnetic dipole moment is defined as m=χHV, where χ is the volume susceptibility and V the volume[11]. The divergence from magnetization equilibrium gives rise to magnetic anisotropic energy, which is solely dependent on the dephase angle, given that the induced magnetic moment inside the rods remains constant. For a thin and flat nanorod, this energy can be simply expressed as

$$E = \frac{1}{2}\mu_0 m^2 sin^2\theta, \tag{9}$$

where μ_0 is the magnetic permeability of free space, m is induced a magnetic moment of the nanorods in an external magnetic field, and Θ is the angle between long axis of nanorods and direction of external magnetic field[12, 13]. The energy is plotted by Matlab and shown in **Supplementary Fig. 8a**. The magnetic anisotropy energy is featured by two deep 3D potential wells, which tends to trap the magnetic nanorods with their long axis parallel to the direction of external fields.

For AuNRs with arbitrary orientation, which can be denoted by the ket |α, Θ> in **Supplementary Fig. 7a**, their longitudinal excitation state can be expressed as

$$|A> = P|\psi_L> = A_L cos\alpha|z>. \tag{10}$$

Plotting this equation (**Supplementary Fig. 8b**) in the Cartesian coordinate by Matlab gives rise to the 3D distribution of longitudinal excitation. In terms of transverse mode, the excitation state is complementary to the longitudinal mode with a sine dependence on the azimuthal angle, α, and plotted in **Supplementary Fig. 8c**. For the cAuNRs, which feature longitudinal concave parallel to the log axis of the nanorods, the surface concave creates another transverse mode and breaks the symmetry of classical one. Considering the rotational freedom of cAuNRs, another angle (δ), in addition to the two azimuthal angles (α and Θ), should be introduced to describe the

excitation of the second transverse mode. Explicitly, it is defined as the angle between the line connecting the center of AuNRs and magnetic nanorods and oscillation direction of the magnetic field when the wave vector of light is parallel to the long axis of nanorods (**Supplementary Fig. 7a inset**). According to the simulated ECS and SCS in **Supplementary Fig. 7a and 7b**, the intensity of second transverse mode approaches maximum when δ is 0° and minimum when δ is 90°. Similar to the dependence of transverse mode on α, the second transverse mode follows sine function on the rotational angle, γ, as well and can be expressed as

$$|A'_T >= |\psi'_T > = A'_T cos\alpha sin\delta|z >, \quad (11)$$

which is plotted in **Supplementary Fig. 8d**. For cAuNRs, their second transverse excitation is remarkably unique. The concave structure breaks the symmetry of second transverse mode around radius direction, giving rise to maximum scattering at $|0^o>$ configuration.

Another characteristic of the Fe_3O_4/Au hybrid nanorods is the parallel alignment of Au and magnetic nanorods, which couples the corresponding anisotropies to each other. Similarly, when the long or short axis of cAuNRs is parallel to the polarization of light, the corresponding longitudinal or transverse resonance will be excited. Between the two extrema are the superpositions of the two excitation states.

III. Quantifying the correlation between excited states and orientation angles

Analytical resolution: As derived in section II, the dependence of expectation value of absorbance of cAuNRs on orientation angles is expressed as

$$< A_L(\alpha,\theta) >= I_L cos^2\alpha \quad (12)$$

$$< A_T(\alpha,\theta) >= I_T sin^2\alpha \quad (13)$$

for longitudinal and transverse modes, correspondingly. If taking the longitudinal (ϕ_T) and transverse mode angle (ϕ_L) into consideration, we have the following dependence: ϕ_T=90-α and ϕ_L= α. The coefficient I_L and I_T represent the maximum longitudinal and transverse excitation. So, the dependence of excitation on the orientational angle can be simply deduced as:

$$\frac{<A_L(\alpha,\theta)>-<A_L(90^o,\theta)>}{<A_L(0^o,\theta)>-<A_L(90^o,\theta)>} = cos^2\alpha \quad (14)$$

$$\frac{<A_T(\alpha,\theta)>-<A_T(0^o,\theta)>}{<A_T(90^o,\theta)>-<A_T(0^o,\theta)>} = sin^2\alpha \quad (15)$$

and α ϵ [0°, 90°]. The dependence is plotted in **Fig. 2g**.

Modeling simulation: The simulated spectra of cAuNRs under various orientations are computed by finite-element frequency-domain method (Comsol Multiphysics). To explicitly model component diversity and the structural complexity, morphologies and size are all derived from TEM images. The shape of initial AuNRs is derived as a flat rod terminated with two half ellipsoids (**Supplementary Fig. 5**) because the morphology of cAuNRs observed from TEM images is neither perfect rod nor an ideal ellipsoid. Basically, the two ellipsoids on the ends can refine the sharpness and make the model more feasible. To create the concavity in the model, we removed another cutting rod with a radius of 10 nm was built atop the AuNRs, and the overlay of the two. As shown in **Supplementary Fig. 5**, the sharp edge around the concave was further rounded by another rod with a radius of 50 nm to mimic the smooth surface of cAuNRs as evidenced by TEM images in **Supplementary Fig. 2d**. Magnetic nanorod was modeled as a cylinder with large aspect ratio and two spherical ends. The overall size is 110 nm in length and 20 nm in width.

The material domains were defined separately to endow them basic physical properties, like relative permittivity or refractive index. The interpolation functions for the complex refractive index of gold is taken from the Optical Materials Database. The refractive index of surrounding is set as 1.5 to mimic the presence of the polymer shell. A plane electromagnetic wave propagates along the x-axis and is polarized in the z-axis. The wavelength of the electromagnetic wave was swept from 400 nm to 1000 nm with a 10-nm step. The absorption scattering cross section was computed by integrating power loss density over the volume of Au and Fe_3O_4 nanorods. Scattering cross section was defined as integral of the dot product of surface normal vector and Poynting vector over the close surface of both Au and Fe_3O_4 nanorods. The extinction cross section is simply the sum of the two. After computing the scattering field by solving the Maxwell's equations, the electric field norm and Poynting vector at a specific wavelength were plotted to visualize the localized surface plasmon resonance and scattering around the concave structure. Explicitly, the azimuthal angle, ϴ, are set at 90° to get rid of multiple excitation and spin angle, γ, is set at 45°, which is actually the expectation value of random spinning states and therefore constant to the transverse excitation of being measured.

The spectra of cAuNRs under different orientations were simulated by varying azimuthal angle, α, between 0° and 90° with a 15° step. The extinction, absorption and scattering cross section were plotted in **Supplementary Fig. 9a, 9b, and 9c**, correspondingly. To derive the correlation between extinction maximum and azimuthal angle, α, the following expression was derived to plot the simulated transverse and longitudinal excitation in **Fig. 3g**:

$$\frac{E_L(\alpha,\theta)-E_L(90^o,\theta)}{E_L(0^o,\theta)-E_L(90^o,\theta)} = cos^2\alpha \quad (16)$$

$$\frac{E_T(\alpha,\theta)-E_T(0^o,\theta)}{E_T(90^o,\theta)-E_T(0^o,\theta)} = sin^2\alpha \quad (17)$$

Experimental: The geometry for experimental measurements is shown in **Fig. 2a** and the extinction spectra under different orientational angles are plotted in **Supplementary Fig. 10 and 11**. Similar data process was carried out to figure out the correlation between excitation state and orientational angles, which was represented in **Fig. 2g**.

IV. Fixing the orientation of cAuNRs by photolithography

The cAuNRs are first dispersed in ethylene glycol, containing AM, BIS, photoinitiator, and then loaded between the glass substrate and coverslips. The sample is then placed in the center of two identical permanent magnets with uniform field strength of ~25 mT. Afterwards, UV light is used for polymerization and cAuNRs with designated orientation will be fixed in the polymer films. To verify the alignment of cAuNRs along the designated directions, we first examined the extinction spectra of the as-made plasmonic films under various orientations. As shown in **Supplementary Fig. 13**, under 0° configuration when the applied magnetic fields during polymerization were parallel to the polymerization, only longitudinal modes were excited, and the transverse modes disappeared. Accordingly, the film appeared the complementary green colors. When rotating the films from 0° to 90°, the longitudinal modes were suppressed gradually, and instead transverse modes were enhanced. The perceived color of the plasmonic films turned from green to red. The angle-dependent plasmonic excitation of the plasmonic films was compared with the theoretical prediction in the polar plot in **Supplementary Fig. 13**. The extinction of transverse and longitudinal modes coincided well with the predicted values, which experimentally confirmed the parallel alignment of the long axis of cAuNRs to the directions of

applied fields. We further examined the alignment of cAuNRs inside the polymer film by imaging the cross sections of the polymer films under SEM. The SEM images of cAuNRs for the alignment was shown in **Supplementary Fig. 14a**. The cross section of pure polymer film was shown in **Supplementary Fig. 14b**, it exhibited flat surface. In the case of polymer film embedded with cAuNRs, the hybrid nanorods could be clearly observed in the cross section. Notably, the orientation of cAuNRs in **Supplementary Fig. 14c** was random as no magnetic fields were applied during polymerization. To verify the alignment of cAuNRs inside the films under applied fields, we prepared two films. In the first one (**Supplementary Fig. 14d**), a magnetic field was applied along the length of the film. In the second film (**Supplementary Fig. 14e** and **14f**), the magnetic field was applied along the width. The cross sections were prepared by cutting the as-made plasmonic films along their width. It is interesting to point out that only one end of the cAuNRs was observed in **Supplementary Fig. 14d**, indicating the uniform perpendicular alignment of cAuNRs to the cross section. In **Supplementary Fig. 14e** and **14f**, parallel configuration was observed with perfect parallel alignment between cAuNRs. Therefore, the alignment of cAuNRs in SEM images was consistent with the direction of applied magnetic fields. To quantify the orientational order of cAuNRs, we measured the alignment angle of 100 cAuNRs within polymer films, which were made under the absence (control group) and presence (experimental group) of magnetic fields. The probability was plotted against alignment angle in **Supplementary Fig. 14g**. Under the absence of magnetic fields, the orientation is evenly distributed, indicating quite random alignment of cAuNRs. When a uniform magnetic field was applied to align the cAuNRs during polymerization, the orientation probability was significantly narrow with a normal distribution and a standard deviation of 2.80^{o} along the direction of external magnetic fields (0°). We further estimated the orientational order parameter by the equation: $S=2\cos^2\theta - 1$, where θ is the angel between the ling axis of cAuNRs and the direction of the magnetic fields[14]. It was found that the orientational order parameter was 0.992, further indicating the perfect alignment of cAuNRs within the films.

To create pre-designed patterns and images, photomask is placed right above the devices, and then a permanent magnet is applied to orientate cAuNRs in a desirable direction. The transparent parts of photomask enable a high transmittance of UV light to the polymer resin, which will initiate the polymerization and consequently fix the orientation of cAuNRs. By sequential orientation-polymerization process, desirable images can be printed by controlling orientational

dispersion of cAuNRs magnetically. The cAuNRs with three typical configurations, vertical, perpendicular and horizontal orientation, are fixed in different areas. Under ordinary backlit in **Supplementary Fig. 15a**, transverse mode is excited only in the perpendicular configuration area, giving rise to red colors in the boundary and grayish in the middle because of the co-excitation of two modes. A clear image is observed (**Supplementary Fig. 15b**), however, under horizontally polarized light due to the selective excitation of one plasmon mode of cAuNRs orientated orthogonally inside polymer matrix. In the initial state, the areas under horizontal orientation ($|0^{o}>$) of cAuNRs exhibit uniform green colors owing to the exclusive excitation of longitudinal modes, while areas corresponding to vertical orientation ($|90^{o}>$) are red since only transverse mode is excited. If rotating the plasmonic film clockwise by 45° as shown in **Supplementary Fig. 15c**, however, the contrast disappears because the two orthogonal states, $|0^{o}>$ and $|90^{o}>$, collapse into $|45^{o}>$ by a 45° rotating operator, resulting in identical excitation states and colors within the plasmonic film. Further clockwise rotation will induce separation of plasmon resonance again and more specifically if rotating by another 45°, exchange of plasmon resonance occurs between $|0^{o}>$ and $|90^{o}>$ (**Supplementary Fig. 15d and Supplementary Movie 6**). Revealed experimentally is the color exchange of those two areas. To fully take advantages of the direct colloidal synthesis method, we synthesized short cAuNRs with their longitudinal modes at short wavelength and used them to create another pattern with orthogonal orientations in different areas. As shown in **Supplementary Fig. 15e**, vivid blue color was observed when the longitudinal modes were excited. The color of neighbouring stripes switched with one other as the polarization of light changed from vertical to horizontal directions. Notably, the polarization-dependent coloration feature of plasmonic films is well-documented in electrically modulated plasmonics[15], where plasmonic devices change colors in accordance with an external electric field, but rarely reported in magnetically regulated plasmonics previously. On the other hand, it could greatly extend the potential applications of responsive plasmonics considering the contactless, incident yet reversible feature of magnetic interaction.

V. Processing of time- and space-resolved spectra under mechanical perturbation

Pressing perturbation: In the case of pressure, the initial orientation of cAuNRs was described as $|\phi>=|\alpha,90^{o}>$ according to the geometry in **Fig. 2a**. The pressure was applied along the

negative z direction. We used a home-made device (**Supplementary Fig. 16a**) to precisely measure the pressure-induced mechanochromic response of the plasmonic films. This device enables the real-time and *in-situ* monitor of extinction spectra of cAuNRs when the plasmonic films are under different pressures. Basically, the films with cAuNRs aligned along pre-designed directions were sandwiched between two glass slides. Different weight was placed above the device to mimic different pressures. After each addition of weight, the deformation of plasmonic films was balanced for ~15 s before the spectrum was acquired. During experiment, AuNRs were aligned 0° and 30° to the surface normal of the films and their mechanochromic response was systematically studied. As control experiment, plasmonic films with randomly orientated cAuNRs was made under the absence of magnetic fields during polymerization. All the spectra were recorded under ordinary light without additional polarizer. Their extinction spectra under various pressures was recorded. Afterwards, the changes of longitudinal plasmonic extinction was plotted against pressure (**Fig. 3 c, d, and Supplementary Fig. 17**).

Stretching perturbation: In the case of strain, the initial orientation of cAuNRs was described as $|\phi\rangle = |\alpha, 90^o\rangle$ according to the geometry in **Fig. 2a**. The film was stretched along z axis. To measure the mechanochromic response of plasmonic films under various strains, a home-made stretcher was integrated into the in-situ UV-Vis devices (**Supplementary Fig. 16b**). The strain experienced by the plasmonic films can be precisely controlled by the rotating shaft with a minimum stepper movement of 130 µm. During the measurement, a polarizer with a parallel polarization to the stretching direction was applied to monitor the optical response of the plasmonic films. A circular polymer film with horizontal cAuNRs was first made and then clipped to the stretcher. The initial angle (α) between the stretching direction and long axis of imbedded cAuNRs was carefully manipulated before stretching. The extinction spectra of cAuNRs were *in-situ* recorded in a real-time fashion when the strain increased from 0% to 30%. Upon stretching, the angle, α, decreased gradually due to the elastic deformation of polymer films, resulting in the enhancement of longitudinal modes. The mechanochromic response of the plasmonic films with α at each 15° was researched and the changes of longitudinal plasmonic extinction was plotted against applied strain to quantify the correlation between strains and optical signals (**Fig. 3f, 3f, 3g and Supplementary Fig. 18**).

It was found that the plasmonic excitation of cAuNRs was a function of the initial alignment angle, α, and the strain, ε. Based on this fundamental understanding, we proposed the working principle for designing mechanochromic films with unique optical response. As shown in **Fig. 3h**, the plasmonic film was prepared by aligning cAuNRs along two different directions: one along the 45° within the film and another along the perpendicular to the surface. When the film was subjected to the same strain, the plasmonic excitation of cAuNRs would change linearly as ε, resulting in blue colors in the corresponding regions. In the case of perpendicular alignment regions, the color did not change significantly as the change in the plasmonic excitation of cAuNRs was negligible small (**Fig. 3g**). We referred this kind of plasmonic films with pre-designed different orientation of cAuNRs as α-contrast films as the different mechanochromic response was based on the difference in their initial angle, α.

Rotating perturbation: For sensing bending motion, cAuNRs were fixed 45° to the surface normal as illustrated in **Fig. 4a**. Under cross-section view and horizontally polarized light, the mode angle increases if the film is rotated counter-clockwise and decreases if clockwise. In other words, longitudinal excitation will be enhanced while transverse excitation will be suppressed under a negative rotational angle (clockwise). To analyze the change of the excited states, we measured the extinction spectra of the film under different rotating angles and plotted in **Supplementary Fig. 19**. Using the spectra of the plasmonic film at a rotation angle of 20° (mode angle of transverse excitation equals 20°) as background, the mode angle of transverse excitation can be derived from the measured spectra based on the following formula:

$$\frac{E_\alpha - E_{20^o}}{E_{90^o} - E_{20^o}} = \frac{sin^2\alpha - sin^2 20^o}{sin^2 90^o - sin^2 20^o} \quad (18)$$

where E_α indicates the maximum transverse extinction of spectra measured at the rotating angle of α. The ϕ_T indicates the derived mode angle of transverse excitation corresponding to rotational angle, α, and was plotted in **Fig. 4d**. A simple rearrangement of Supplementary Equation 18 gives rise to another important expression:

$$\phi_T = 90^o - \alpha = 90^o - \arcsin\left(\frac{E_\alpha - E_{20^o}}{E_{90^o} - E_{20^o}} * (sin^2 90^o - sin^2 20^o) + sin^2 20^o\right)^{0.5} \quad (19)$$

Bending perturbation: The only difference between rotating and bending is that, whereas the former one induces a pure change of excited states, the later one gives rise to separation of

plasmon resonance upon bending axis. When the bending angle is set at α, the term turns to be 45°+α for the left end and 45°-α for the right end. Therefore, the expectation value of transverse excitation for left and right end can be expressed as $\cos^2(45^o+\alpha)$ and $\cos^2(45^o-\alpha)$, correspondingly and plotted in **Supplementary Fig. 20**. As bending angle increases, transverse excitation will be enhanced for the left end and suppressed for the right end.

Twisting perturbation: In the case of either rotating or bending, the mode angle of excited states (both transverse and longitudinal mode) is linearly dependent on the angle of mechanical perturbation. The twisting perturbation, however, induces much-complicated behavior of the two excited states across the whole plasmonic film, which can be classified as a nonlinear perturbation. Based on discussion of bending and rotating, it has been recognized that symmetry-breaking of orientation of cAuNRs along the active axis of mechanical motions induces the separation of excited states of plasmonic film upon mechanical motions (**Supplementary Fig. 21**). The twisting effect can be decomposed into localized rotational perturbation with a continuously increased angle, notated as γ, with the formula of ω*y/L, where ω is the overall twisting angle, y is the position of interest and L is the total length of the films (**Supplementary Fig. 22**). The localized rotation will, on the other hand, induce a change of the excited state of plasmon resonance as the angle between long axis of cAuNRs and polarization direction non-linearly and periodically switch with respect to localized rotational angle. The connection between can be derived by solving the geometric correlation as following:

$$\sin \phi_T = sin^2(\frac{\omega * y}{2 * L}) \tag{20}$$

for transverse excitation, where ϕ_T is the transverse mode angle. And, the expectation values for them can be described as following based on bra-ket theorem:

$$< A_T >=< \phi_T|\mathrm{P}|\phi_T >= cos^2\phi_T = 1 - sin^4(\frac{\omega * y}{2 * L}) \tag{21}$$

This prediction was plotted in **Fig. 4h**. Based on this equation, the correlation between γ-ϕ_T can be expressed as:

$$\phi_T = \arccos^{0.5}(1 - sin^4(\frac{\gamma}{2})) \tag{22}$$

To analyze the change of excited states of cAuNRs under nonlinear twisting perturbation, we measured the extinction spectra under a various position of the film and plotted in

Supplementary Fig. 23a, 23b and 23c. To derive the nonlinearity between mode angle and localized rotational angle induced by twisting, we used the maximum extinction of each spectrum at transverse resonance frequency for the following data processing to derive normalized transverse extinction at the location, y (with a localized rotational angle of γ =ω*y/L):

$$\frac{E_x - E_{L/2}}{E_0 - E_{L/2}} \tag{23}$$

The behavior of plasmonic excitation of cAuNRs inside the film under nonlinear twisting perturbation can be visualized by plotting the deduced transverse extinction against localized rotational angle (ω*y/L). As shown by the discrete black dots in **Fig. 4h**, it has a great agreement with the predicted curve represented by the red line. The non-linearity of twisting perturbation is evidenced by plotting transverse mode angle (ϕ_T) versus localized rotational angle in **Supplementary Fig. 23d**. The mode angle can be derived as:

$$\phi_T = arccos(\frac{E_x - E_{L/2}}{E_0 - E_{L/2}})^{0.5} \tag{24}$$

with ω=360° and the localized rotational angle, γ, can be written as:

$$\gamma = \frac{\omega * y}{L} \tag{25}$$

VI. Fabricating Programmable mechanochromic devices

After systematic study of the plasmonic excitation in response to various mechanical motions and deformations, we further demonstrated that magnetic alignment in combination with photolithography can produce mechanochromic devices with pre-designed optical outputs in a programmable fashion. As shown in **Fig. 5a**, patterns were created by magnetically aligning cAuNRs into two different directions. Under the rotating angle of -45°, the longitudinal excitation angle was 90° and 0° in the red and blue regions, respectively. Sine the longitudinal plasmonic excitation can be predicted by cos^2-function, the longitudinal mode in red regions will be suppressed to the minimum while, in the blue regions, it will be enhanced to the maximum, which is quite consistent with the perceived complementary colors in the patterned films. As the rotating angle increases (from -45° to 45°), the longitudinal excitation angle decreases in the rhombus regions and increases accordingly in the remaining regions. Consequently, the rhombus

regions turn from red to gray and then blue. Meanwhile, the remaining areas appears from blue to gray and finally red colors. Overall, the regions with identical nanorod orientation will switch from their initial colors to the counterparts when rotating the film from -45° to 45°. The mechanochromic response to bending is more complicated compared to the case in rotating. As shown in the first image (i) in **Fig. 5b**, the regions with identical rod alignment will exhibit two different colors, among which one (red) is complementary to transverse mode and another (blue) to longitudinal mode, due to the bending-induced separation of plasmonic excitations. As bending angle increased (from backwards to forwards), the initial colors turned to their counterparts with the mechanochromic mechanism as same as rotating. This fabrication process can be easily scaled up to around several centimeters as shown in **Supplementary Fig. 26** and **Supplementary Movie 4**. The films exhibited uniform and clear mechanochromic response to bending and rotating, which further demonstrated the feasibility of the proposed fabrication process.

To demonstrate the real-world applications, we proposed a prototype of mechanochromic devices. As shown in **Fig. 5c**, a PDMS chamber was sealed above a glass slides, the top of which was only covered by a thin layer of PDMS and the as-made plasmonic films. The inner pressure can be controlled by the vacuum valve on the left. When air is extracted from the chamber, the top film is subject to negative pressure and hence bended downwards. When additional air is injected into the chamber, the positive pressure will expand the top thin elastic films in an upwards fashion. Notably, the change of the orientation of cAuNRs within the films is quite different in the two cases. To confirm this hypothesis and verify the mechanochromic response, a butterfly pattern with uniform alignment of cAuNRs was fabricated. In **Fig. 5d**, cAuNRs were all aligned 45° to the surface normal. On its flat configuration (no pressure), both the transverse and longitudinal modes were excited, which gave rise to a gray butterfly. When air was injected to the chamber, the induced positive pressure bended the top film and the orientation of cAuNRs separated into horizontal (left end) and vertical (right) manners. Under a horizontal polarizer, the left wing of the butterfly appeared blue (complementary to longitudinal mode) while the right wing exhibited red (complementary to transverse mode). In the case of negative pressure, the butterfly pattern exhibited inverse coloration. Based on similar working principle, the coloration and patterns during mechanical deformation can be programmed into desirable fashion. As shown in **Supplementary Fig. 27a**, the alignment of cAuNRs was patterned into specific forms.

Under vertically polarized light, the film exhibited red color under pressures as only transverse mode was excited. In the case of horizontal polarization, no obvious pattern was observed as both transverse and longitudinal modes were excited in absence of pressure (**Supplementary Fig. 27b**). When subject to positive pressure, the squares exhibited blue on the left and red on the right parts. Interestingly, the initial colors turned into their counterparts when the pressure switched from positive to negative. The asymmetric mechanochromic response of the plasmonic films provides a novel approach towards next-generation mechanochromic devices capable of identifying the nature of mechanical perturbations. In addition, the plasmonic films can serve as colorimeter to estimate the applied mechanical deformations. Taking pressure for example, as shown in **Supplementary Fig. 27c,** the number and contrast of squares appearing coloration can be used to roughly estimated the applied pressure of the devices. Under lower pressure, only two rows of squares were observed with low contrast to their surroundings. As pressure increased, both the number and the contrast of the squares increased gradually. This readable coloration changes can be potentially engineered into a pressure colorimeter. It is interesting to point out that the mechanochromic films enabled by magnetic alignment of cAuNRs within the regime of anisotropic plasmonics can exhibit asymmetric and programmable mechanochromic response to universal mechanical perturbations by carefully engineering the orientation of cAuNRs in the specific regions of the as-made elastic substrates. As a result, it not only quantitatively provides angular and strain information but also indicates the directions of mechanical perturbations (**Supplementary Movie 7**). To conventional systems, it is still challenging or very difficult to achieve based on the traditional mechanochromic response, such as opal and inverse opal structures based on diffraction or mechanochemochromic materials.

Supplementary Figures

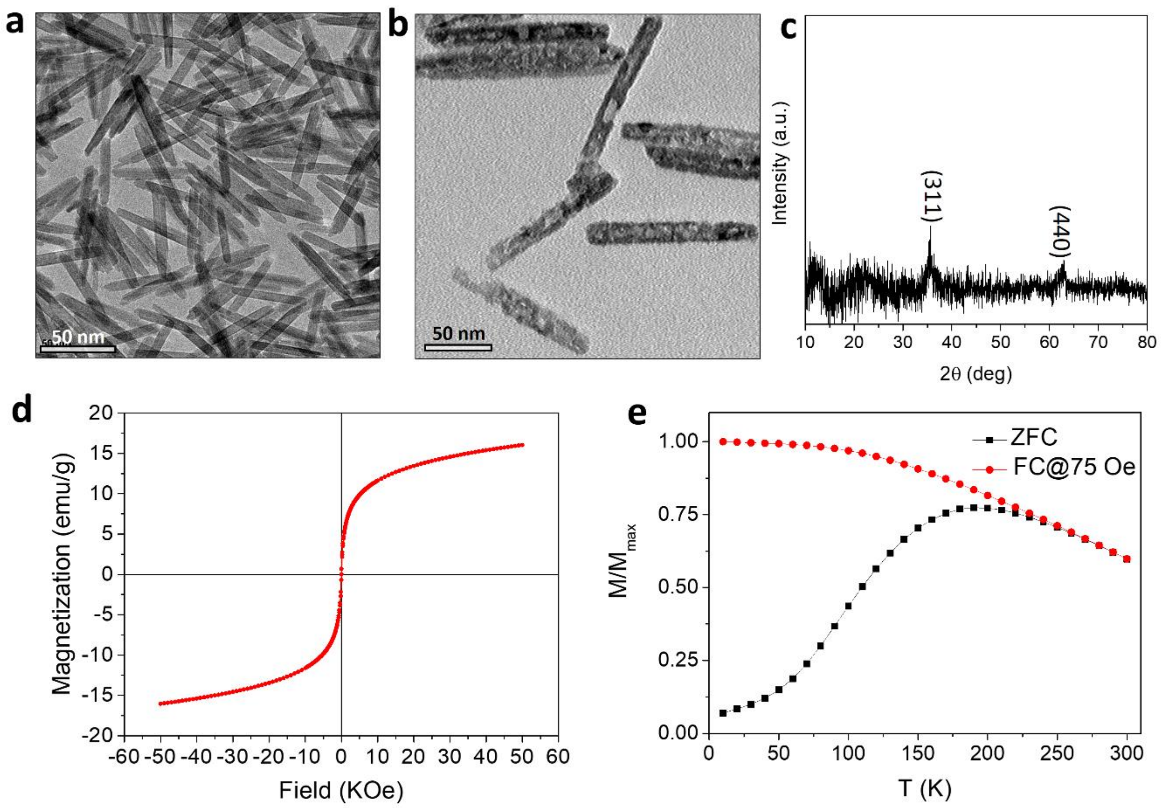

Supplementary Figure 1. TEM images of **(a)** FeOOH and **(b)** Fe_3O_4 nanorods. **c,** XRD pattern of Fe_3O_4@SiO_2 nanorods. **d,** The magnetization curve of Fe_3O_4@SiO_2 nanorods at 300 K. **e,** The ZFC/FC curve of Fe_3O_4@SiO_2 nanorods.

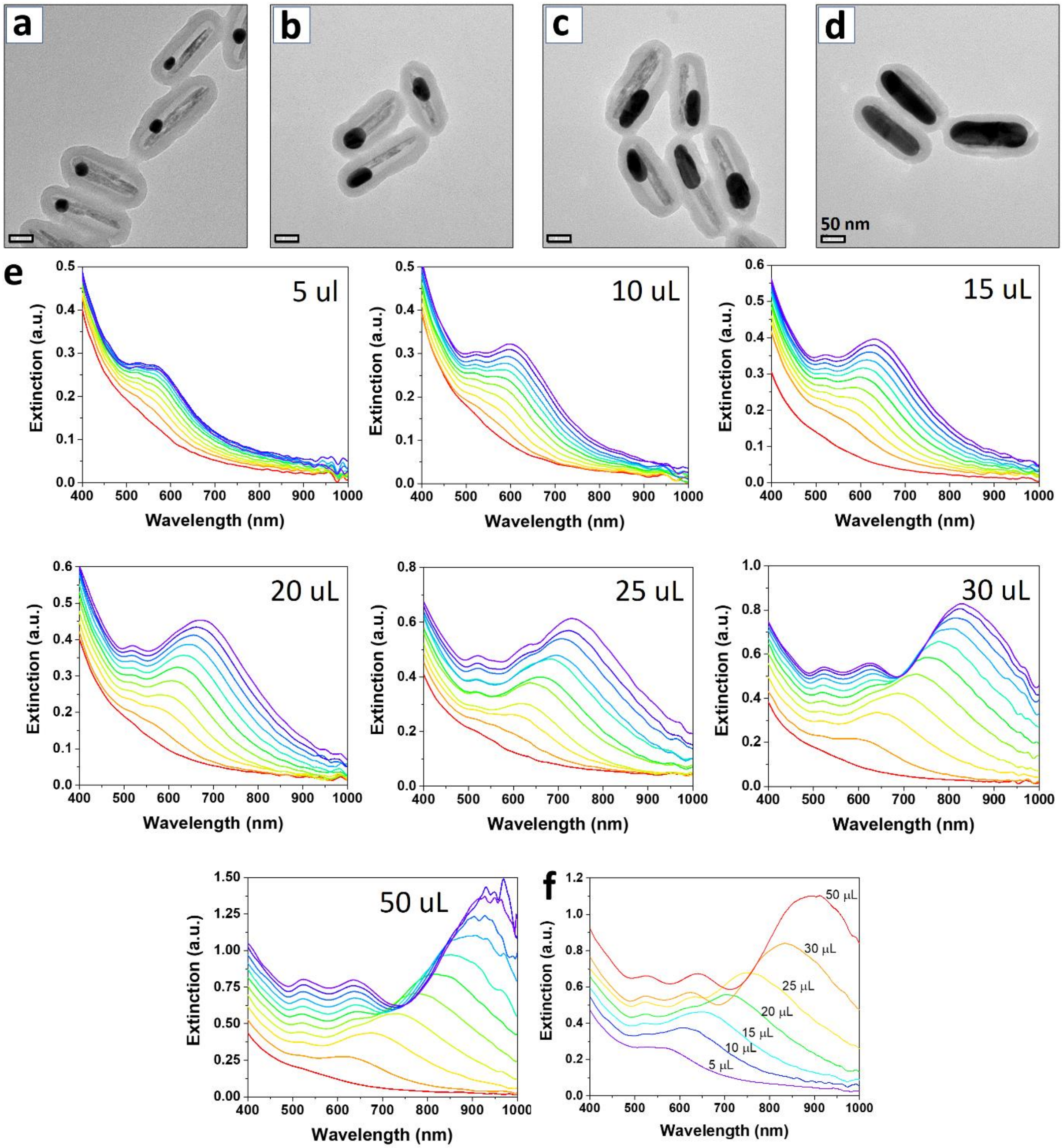

Supplementary Figure 2. TEM images of concave AuNRs prepared by adding **(a)** 5 uL, **(b)** 15 uL, **(c)** 25 uL and **(d)** 50 uL of 0.025M $HAuCl_4$. **e,** Extinction spectra evolution during seeded growth by adding different amounts of $HAuCl_4$ as indicated. All the spectra were measured each 30 s within the total growth time of 5 min. **f,** Summary of extinction spectra of cAuNRs synthesized by adding different amounts of precursors.

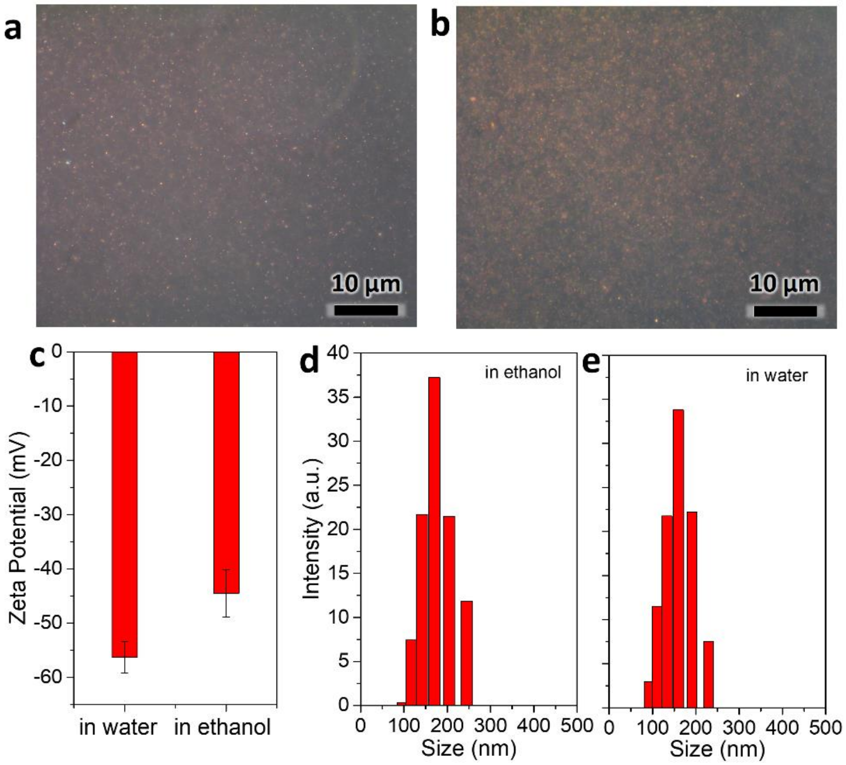

Supplementary Figure 3. Dark-field optical microscopic images of cAuNRs dispersed in (**a**) water and (**b**) EG. **c**, The zeta-potential of cAuNRs dispersions in water and ethanol. Error bars represent the standard deviation from three experimental measurements of the Zeta potential. The size distribution of cAuNRs in (**d**) ethanol and (**e**) water measured by dynamic light scattering.

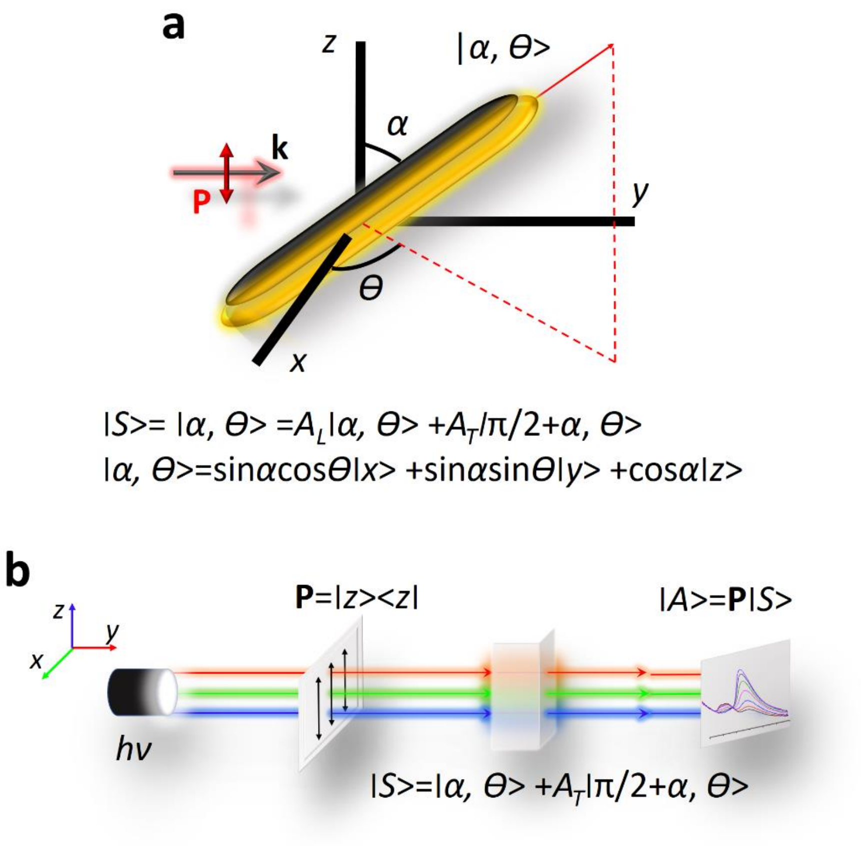

Supplementary Figure 4. Bra-ket notation of linear polarization. a, Schematic illustration of a hybrid nanorod with an arbitrary orientation relative to polarized light incidence. **b**, Schematic illustration showing the plasmonic excitation under linearly polarized light. The incident light is polarized along y axis. The linear polarization can be notated as a project mathematically, |z><z|. To study the tunability of cAuNRs, they are rotated in x-y and x-z plane. In each plane, Θ and α are defined as the angle between x axis and the longitudinal axis of nanorods respectively. In this scenario, the phase of cAuNRs can be interpreted as |α, Θ>. A_L and A_T correspond to the longitudinal and transverse magnitude. Each phase term is composed of three individual and orthogonal vectors. The interaction between polarized light and aligned nanorods can be interpreted mathematically as a projector or operator, *P (*|z><z|*)*, operates on a ket, *|S>*.

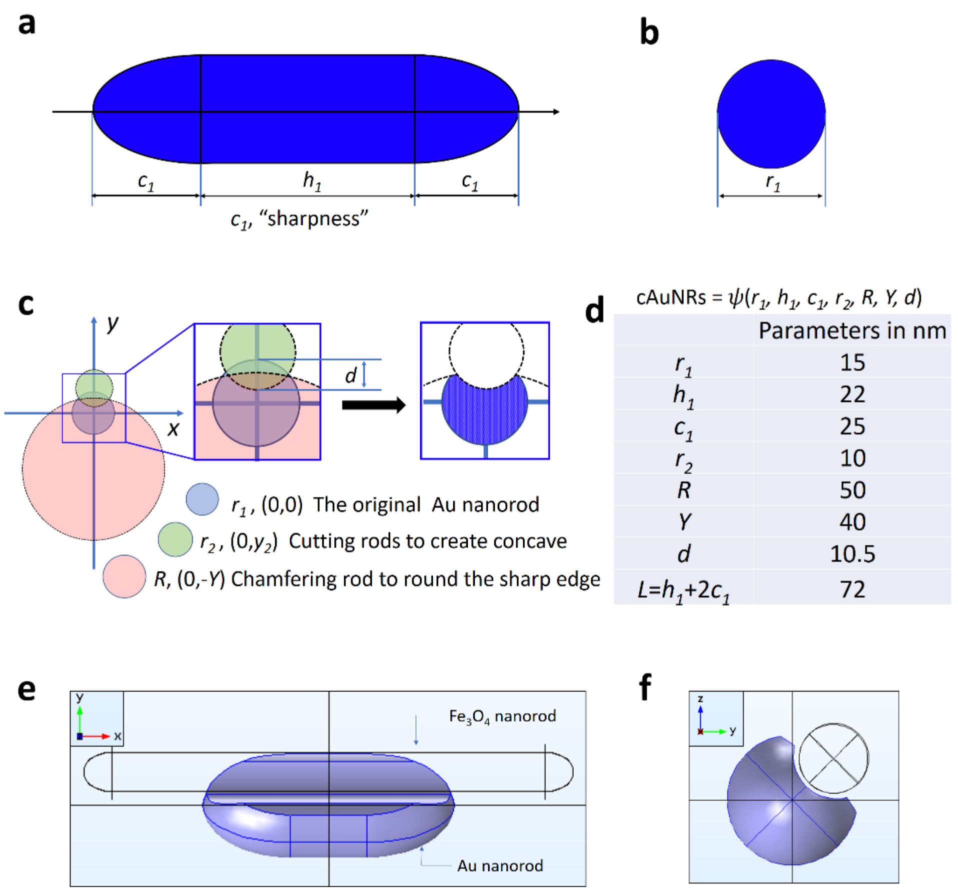

Supplementary Figure 5. **Modeling of cAuNRs.** Longitudinal (**a**) and transverse cross section (**b**) of initial AuNRs. **c**, Illustration in transverse cross-section view of geometry creating and defining the concave structures on the surface of initial AuNRs. **d**, Refining the parameters that determine the cAuNRs. Basically, the morphology of cAuNRs is the function of variables, parameters including radius of initial AuNRs (r_1), cutting rod (r_2) and chamfering rod (R), the length of flat middle part (h_1) and sharp ends (c_1), y-coordinate of center of chamfering rod (R) and concave depth (d). **e**, Multi-component model for Fe_3O_4/cAuNRs nanorods in the longitudinal cross-section view. **f**, The transverse cross-section of Fe_3O_4/cAuNRs nanorods. The blue nanorod represent cAuNRs and long, thin rod-like frame indicates Fe_3O_4 nanorods. The azimuthal state, including both orientation and spin phases, can be notated as |90°, 0°, 45°>. Whereas the orientation state is referring to Fig.3a, the spin is deduced based on Fig. 2a inset. All parameters used in the model are derived from TEM images.

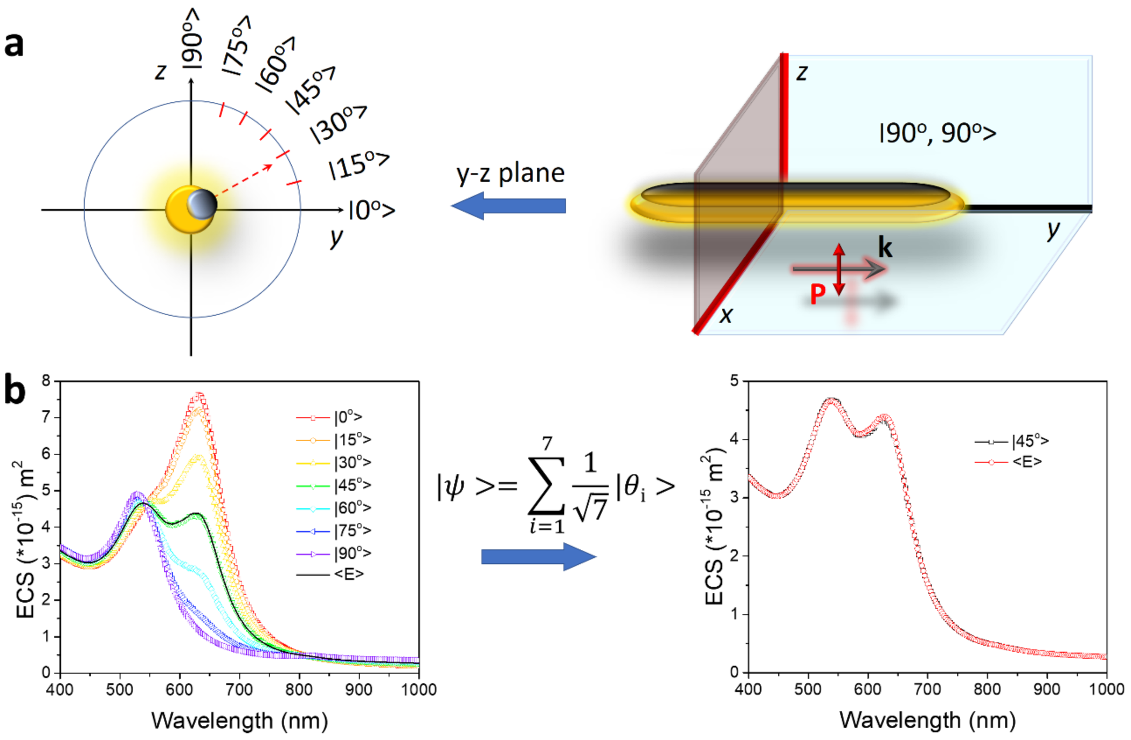

Supplementary Figure 6. Deriving the expectation value of transverse mode based on simulation. Once nanorods are parallel to y-axis, only transverse mode will be excited. The concave on the nanorods creates two transverse modes at different wavelength. To get numerical solution from simulation, seven representative angles in first quadrant is highlighted as shown above. Therefore, the state function of nanorods can be given as $|\psi\rangle$. **a,** Seven chosen states are used to mimic experimental transverse excitation. **b,** The corresponding transverse extinction cross section. <E>, <A> and <S> indicate expectation values (average) of extinction, absorption and scattering, correspondingly.

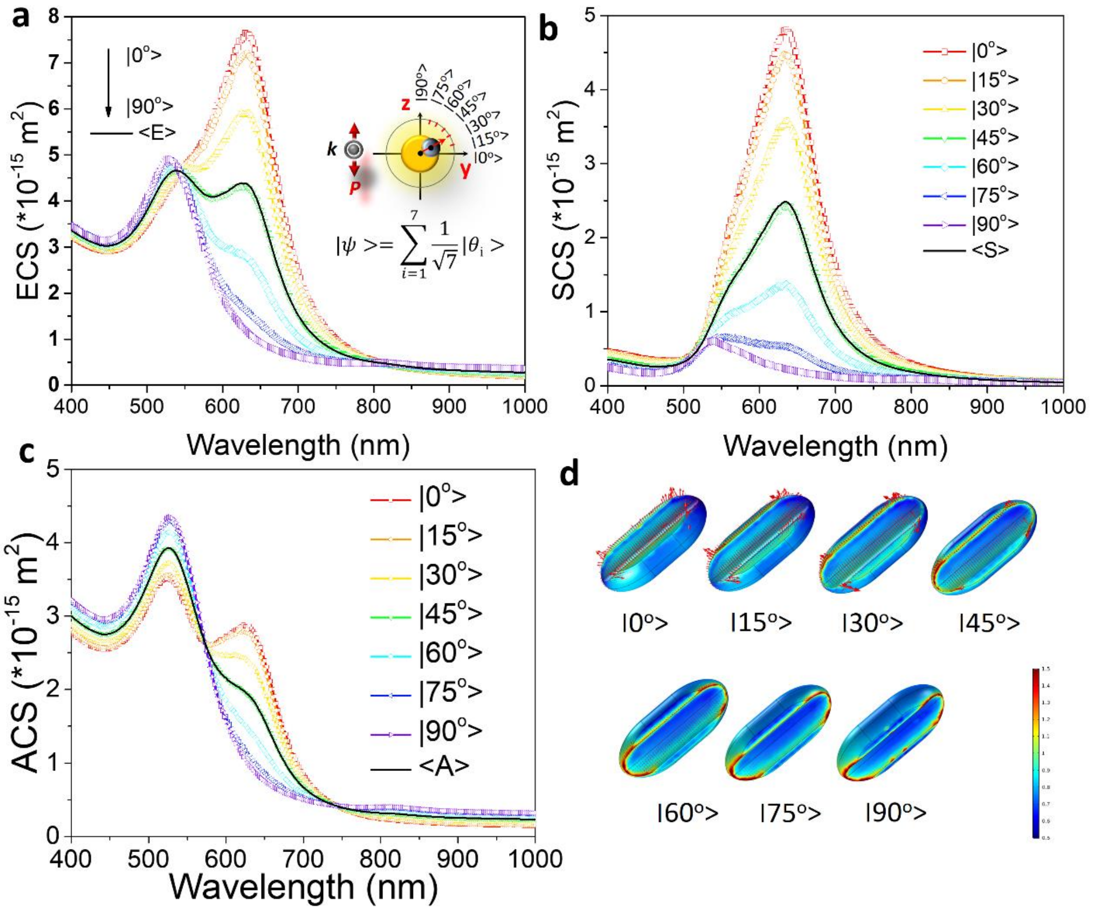

Supplementary Figure 7. a, Simulated transverse extinction cross section (ECS) of cAuNRs. Inset: Geometry defining excitation states of transverse plasmonic mode, where k indicates the wave vector and p directs polarization direction. **b,** Simulated scattering cross section (SCA) of cAuNRs under different orientational configurations. **c,** Simulated absorption cross section (ACS). **d,** Electric filed distribution and Poynting vectors, *S*, on the surface of cAuNRs under excitation wavelength of 630 nm. Inset: Geometry defining excitation states of transverse plasmonic mode. <E>, <A>and <S> are the expectation values (average) of extinction, absorption and scattering, respectively.

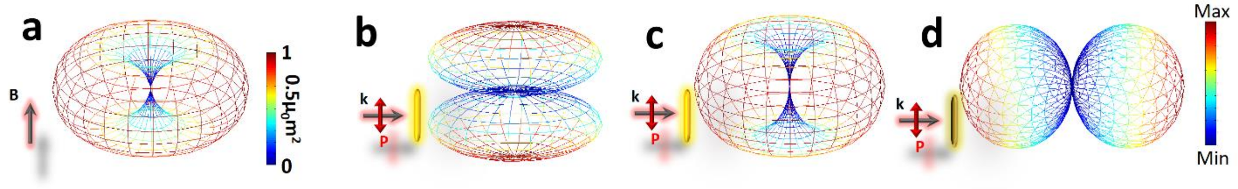

Supplementary Figure 8. a, 3D plots showing magnetic anisotropy of magnetic nanorods. The induced magnetic moment is denoted as m and μ_0 is the permeability constant (permeability of free space). 3D distribution of longitudinal **(b)** and transverse **(c)** excitation of plasmonic modes of AuNRs. **d,** Excitation of second transverse mode of cAuNRs. The concaved structures break the symmetry of transverse excitation. **b**, **c**, and **d** share the same color scale bar.

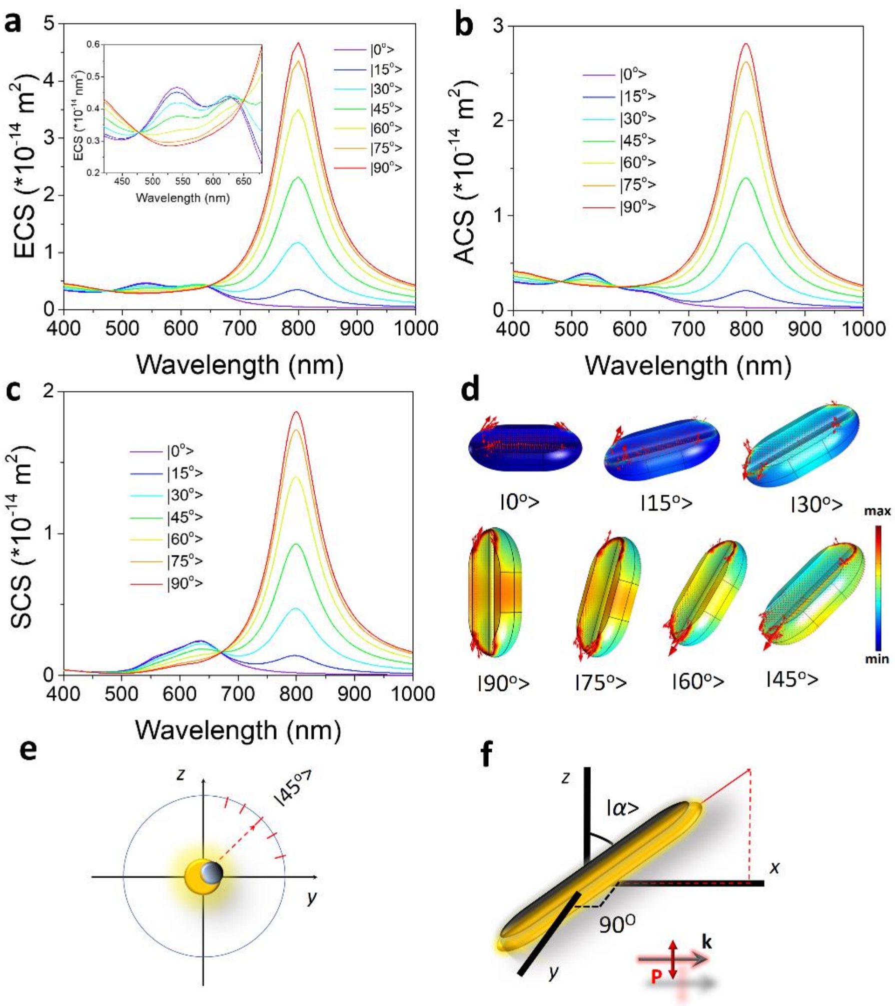

Supplementary Figure 9. Simulated extinction **(a)**, absorption **(b)** and scattering cross section **(c)** of cAuNRs under different orientations. Inset in **(a)** shows the transverse tunability under different orientations. **d,** Surface distribution of electric field under different orientations at excitation wavelength of 800 nm. Surface arrows indicates localized Poynting vectors at excitation wavelength of 630 nm. **e,** To mimic the random excitation of two transverse modes, the concave structure is orientated at 45° in z-y plane base on the assumption that random dispersion gives rise to equal excitation of the two modes, which is evidenced in Supplementary Fig. 6. **f,** The cAuNRs are re-orientated from x-axis to z axis. Spectra are taken each 15° to simulate the transition from transverse excitation state to longitudinal excitation state.

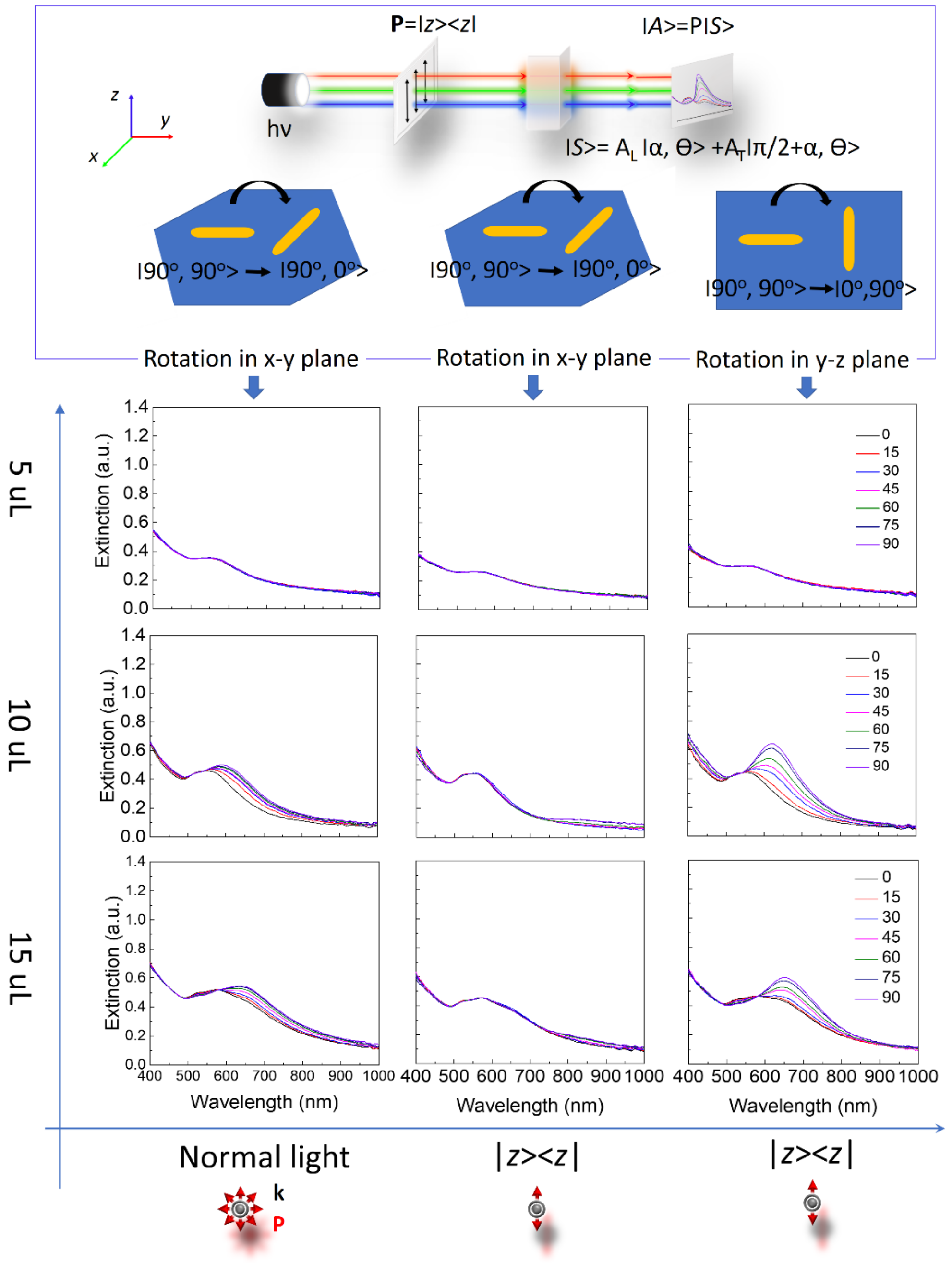

Supplementary Figure 10. Tuning plasmonic extinction of concave AuNRs under polarized light and its mathematical interpretation. From top to bottom, the volume of precursor increases gradually as indicated. Extinction spectra on left panel was measured under ordinary light, while middle and right panels are measured under z-polarized light. All the spectra share the same figure legend.

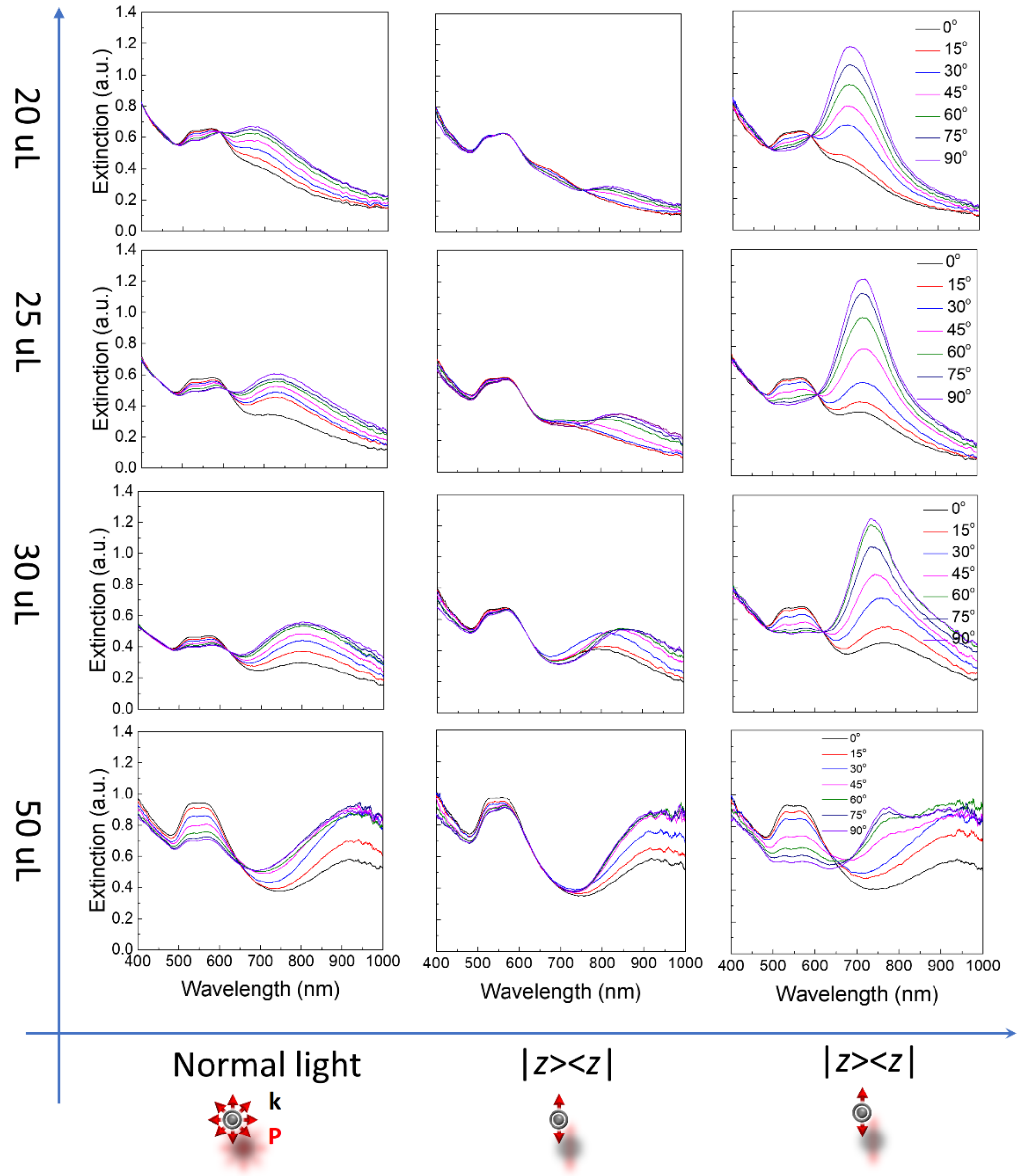

Supplementary Figure 11. Continued extinction spectra from **Supplementary Fig. 10.**

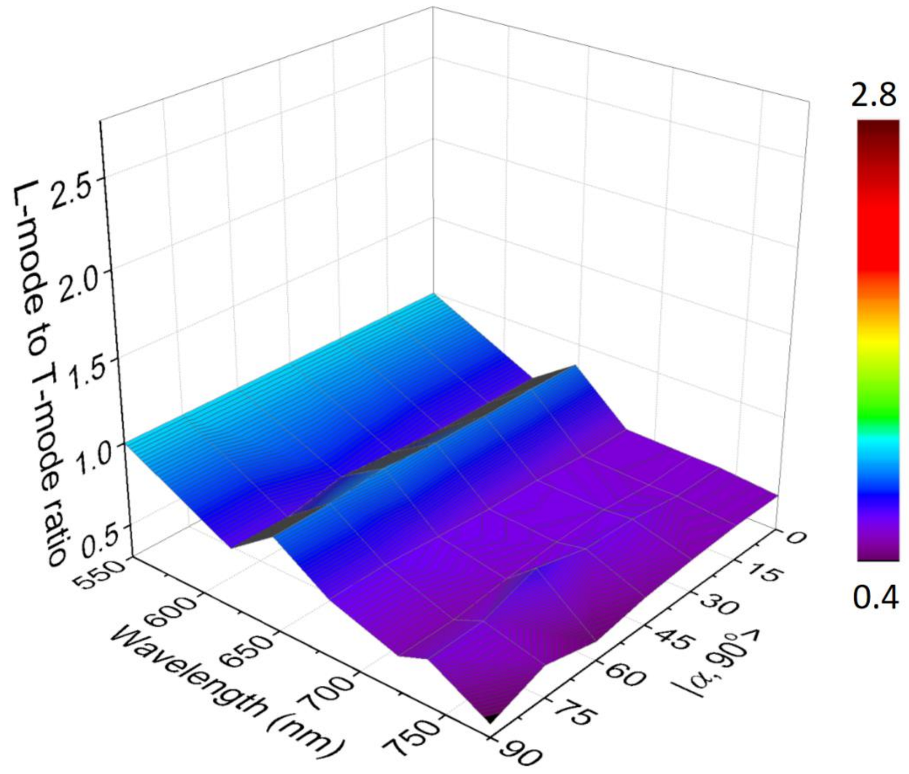

Supplementary Figure 12. Tunability factor of cAuNRs with different aspect ratio characterized by peak position of longitudinal absorption under polarized light. The L-mode to T-mode ratios are summarized from the middle panels from **Supplementary Fig. 10 and 11.**

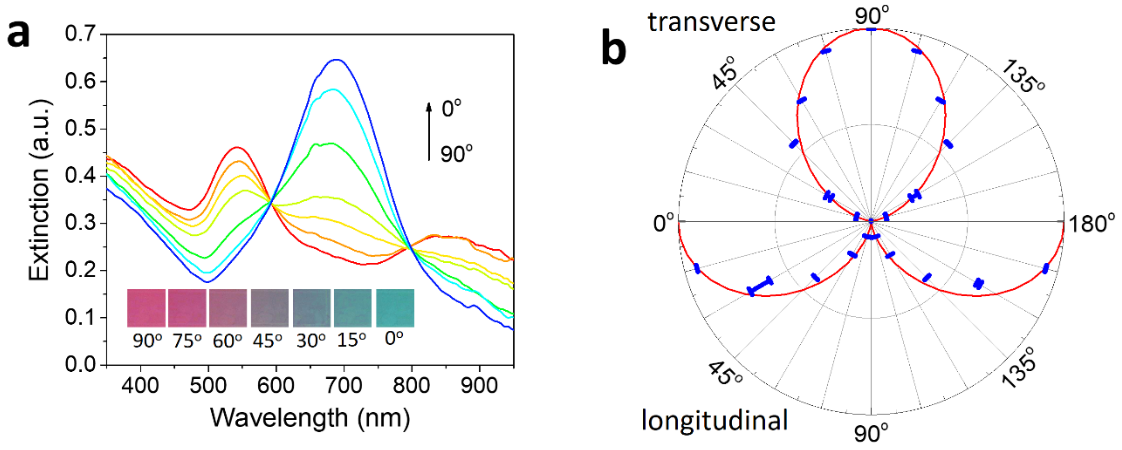

Supplementary Figure 13. a, Extinction spectra of plasmonic film at different rotational angles. Inset shows its corresponding colors. **b,** Polar plot of transverse and longitudinal modes of cAuNRs in solid plasmonic films. Error bars represent the standard deviation from three experimental measurements of the extinction spectra under different magnetic fields. Blue dots represent the measurement values and red lines indicates the theoretical predictions. The good agreement between the experimental measurements and the theoretical prediction demonstrates the perfect alignment of cAuNRs along the applied magnetic fields.

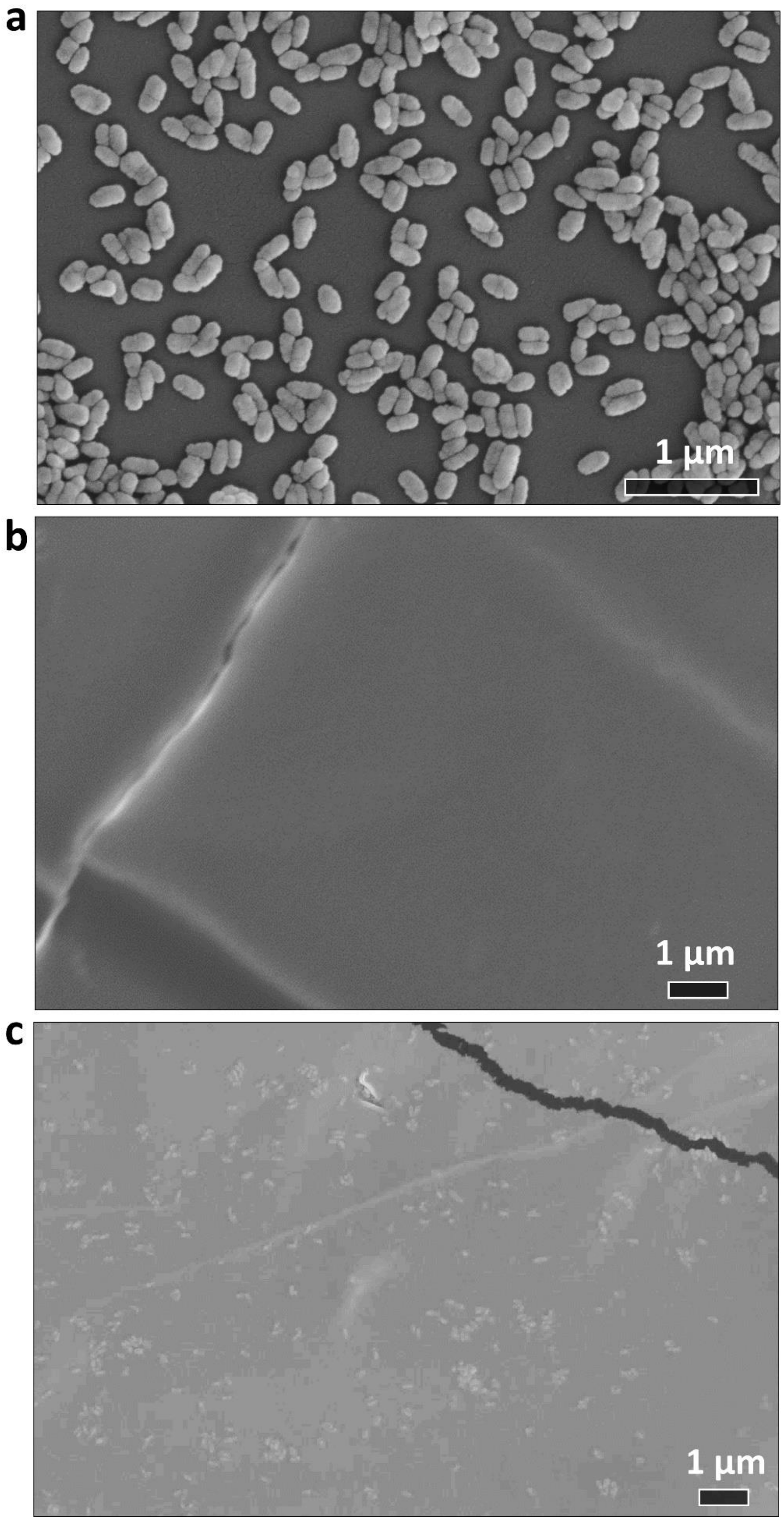
a
1 µm
b
1 µm
c
1 µm

d

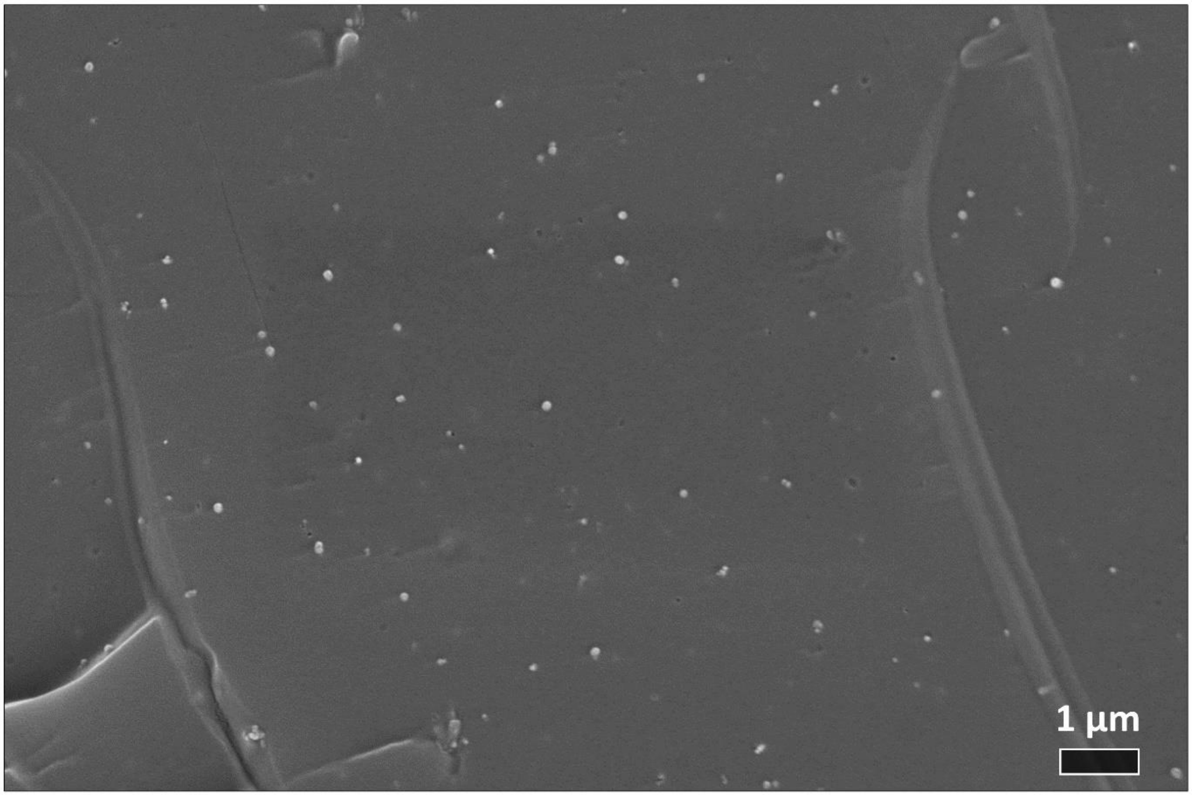

e

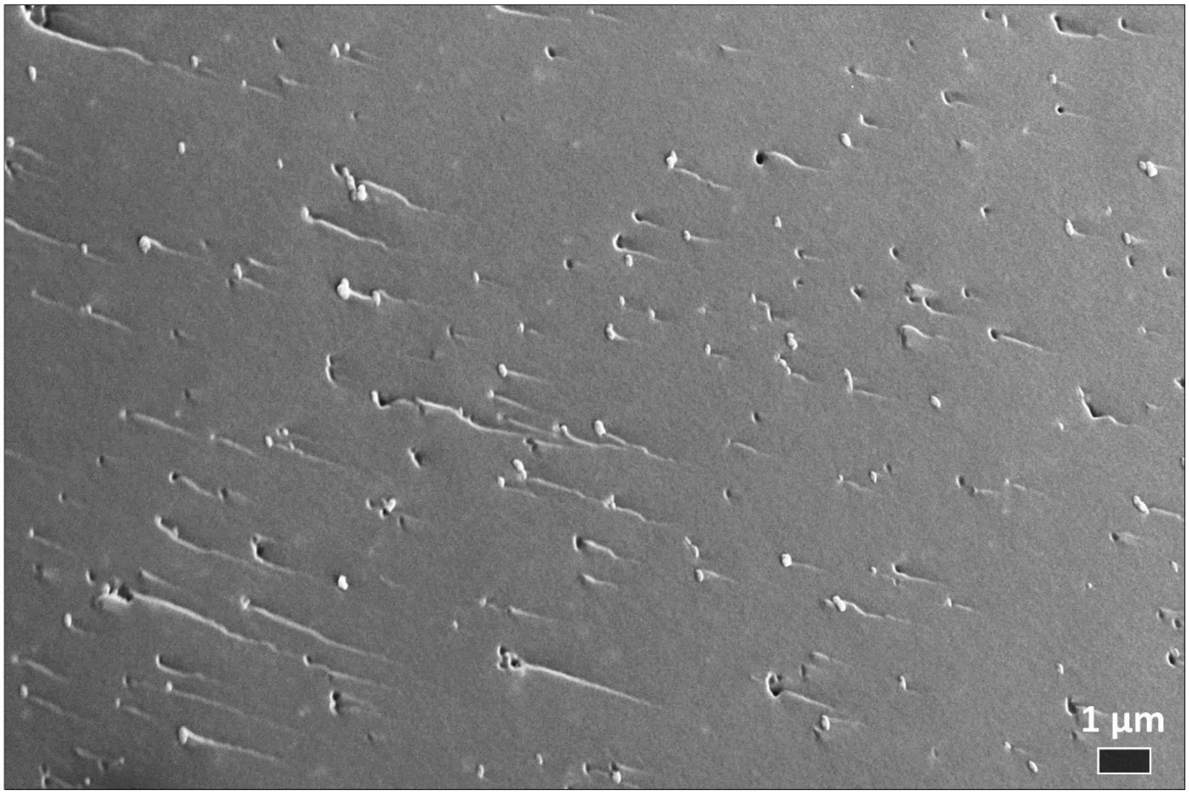

f

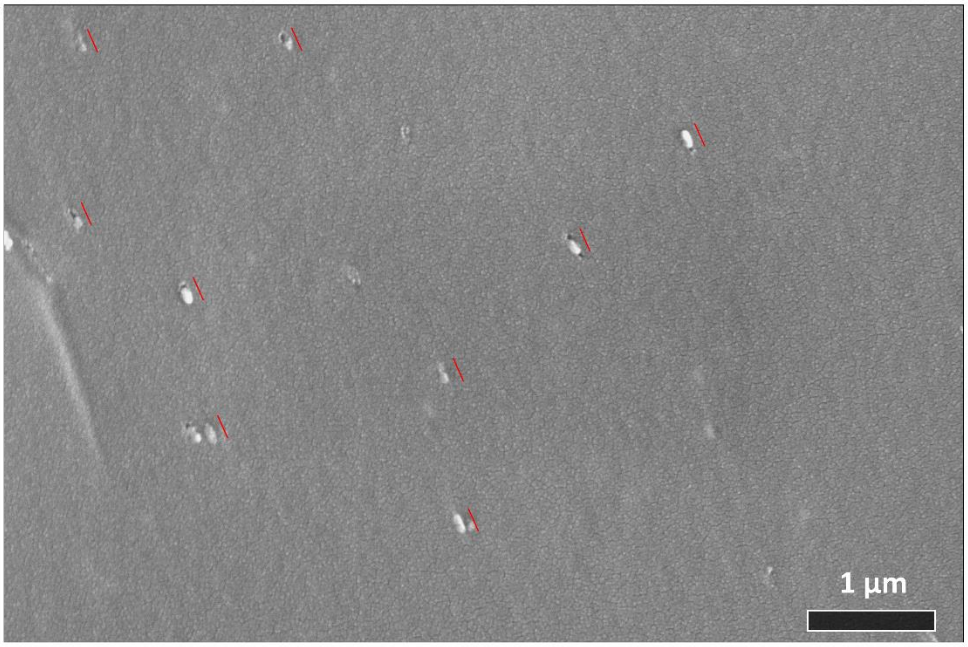

g

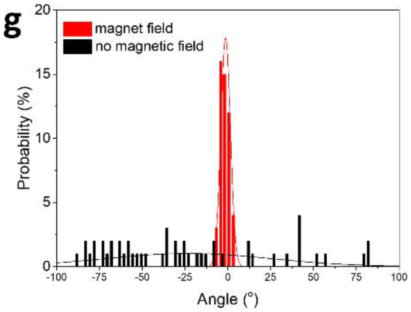

Supplementary Figure 14. a, SEM image of the hybrid Fe_3O_4/Au@RF nanorods. SEM images of cross sections of PAM hydrogels **(b)** without hybrid nanorods, **c-f,** embedded with hybrid nanorods. In **c**, the orientation of cAuNRs is random as no magnetic fields were applied during polymerization. The polymer films were prepared by applying magnetic fields along the length in **d** and width in **e, f** of the films. Cross sections were prepared by cutting the hydrogels into thin films along their width. The perpendicular and parallel alignment of cAuNRs to the surface of the films indicated the good orientational order of cAuNRs and their parallel alignment along the direction of the applied magnetic fields. In **f**, reference lines are added to indicate the good alignment of cAuNRs. **g,** The probability of cAuNRs orientation inside plasmonic films with and without magnetic fields during polymerization. In each group, the orientation of 50 cAuNRs was measured and plotted by the probability.

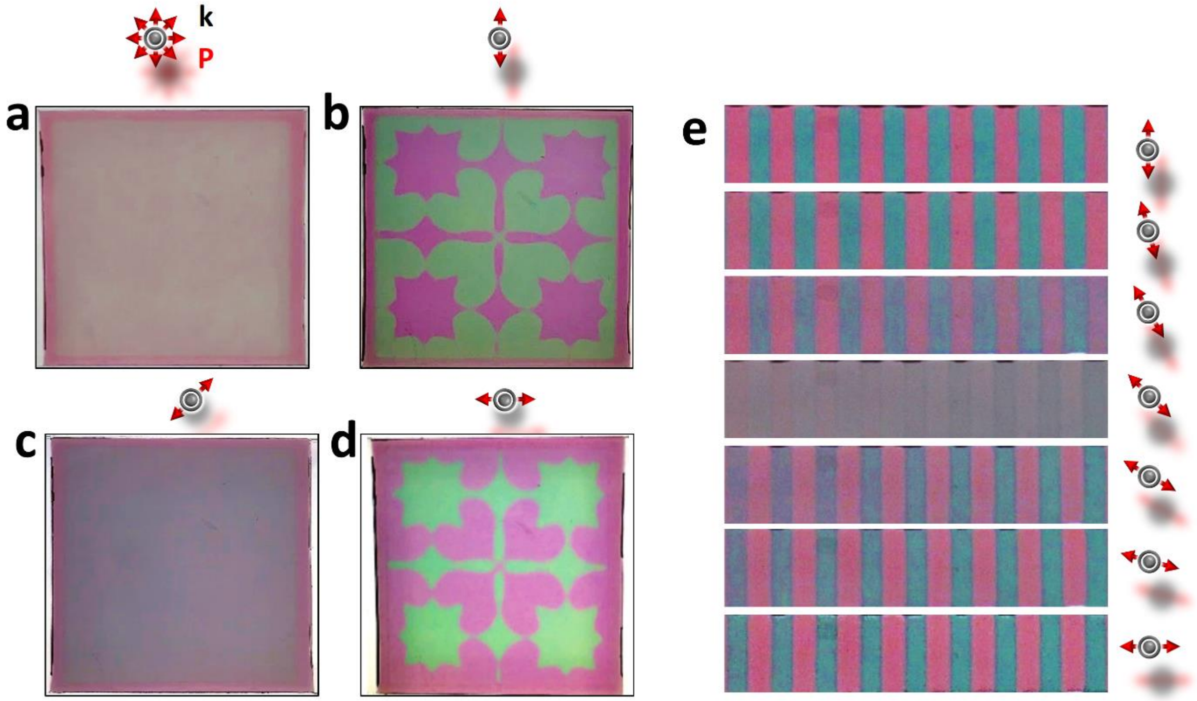

Supplementary Figure 15. Polarization-dependent coloration of plasmonic films by lithography. a, Digital image of the plasmonic film under illumination of ordinary backlit. Digital images of the plasmonic film under illumination of **(b)** vertical polarized light, 45° polarized **(c)** and horizontally polarized light **(d).** Red arrows indicate the polarization direction while gray dots illustrate that the wave vector is perpendicular to the surface of film. In **b**, the orientation of cAuNRs is horizontal in red areas and vertical in green areas. **e,** Colorful stripes of plasmonic films with cAuNRs aligned orthogonally in the separate regions. In the top panel, the orientation of cAuNRs is horizontal in red areas and vertical in blue areas.

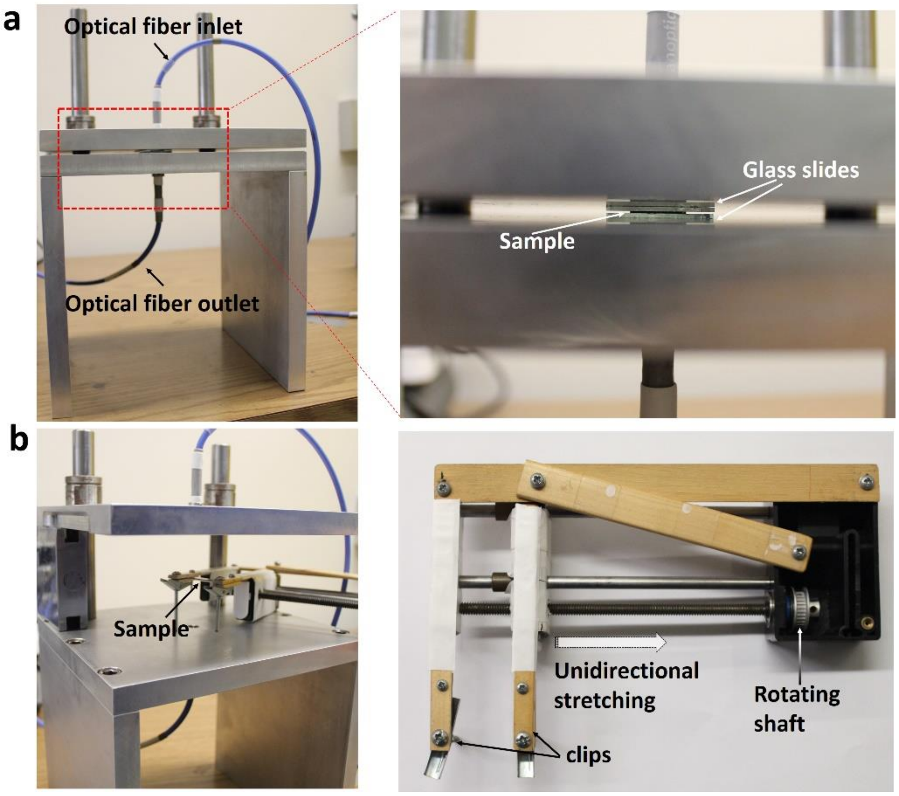

Supplementary Figure 16. Digital pictures of setups for *in-situ* and real-time measurement of **(a)** pressure- and **(b)** strain-induced mechanochromic response of plasmonic films. In **a**, the plasmonic films were sandwiched between two glass slides. Weight can be placed above the samples and the plasmonic excitation of cAuNRs inside the plasmonic films can be measured conveniently.

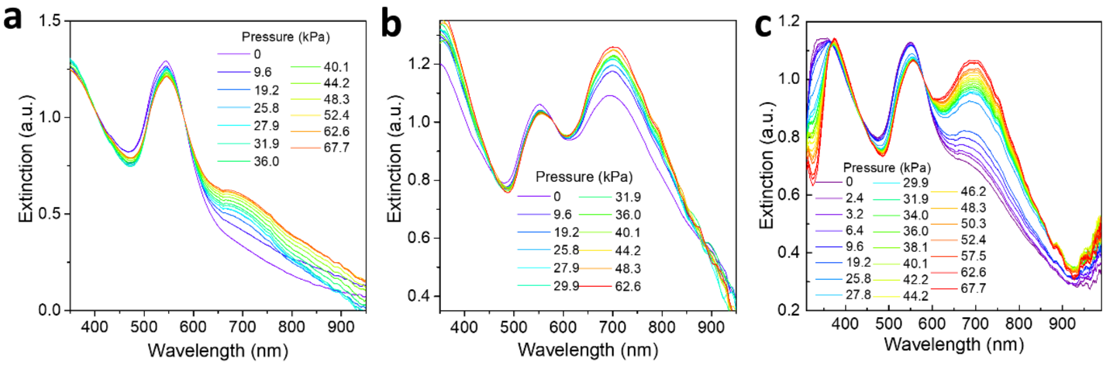

Supplementary Figure 17. Extinction spectra of the plasmonic films under different pressures. **a,** No magnetic field was applied during UV-initiated polymerization. The orientation of cAuNRs is random. **b,** The orientation of cAuNRs was magnetically aligned parallel to the surface normal of the plasmonic film. **c**, The orientation of cAuNRs was magnetically aligned 30° to the surface normal of the plasmonic film.

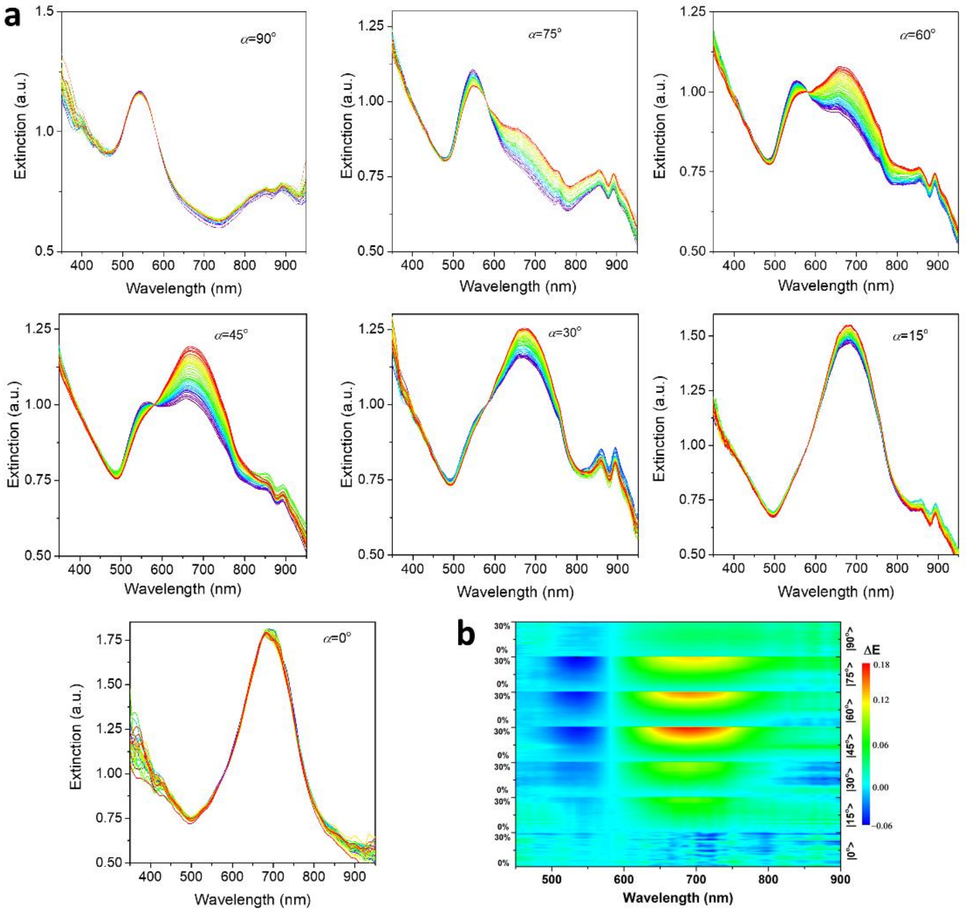

Supplementary Figure 18. a, Extinction spectra of the plasmonic films under different strains. The line profiles represented by the rainbow colors from blue to red are the extinction of cAuNRs subjected to an increasing strain from 0% to 30 %, respectively. The stretching angle, α, is defined as the angle between stretching vectors and the long axes of cAuNRs collectively aligned inside the films. **b,** Color contour of intensity changes of the film extinction (ΔE) upon stretching. The stretching angle (labeled on the right y-axis), α, is defined as the angle between stretching directions and the long axes of cAuNRs collectively aligned inside the films.

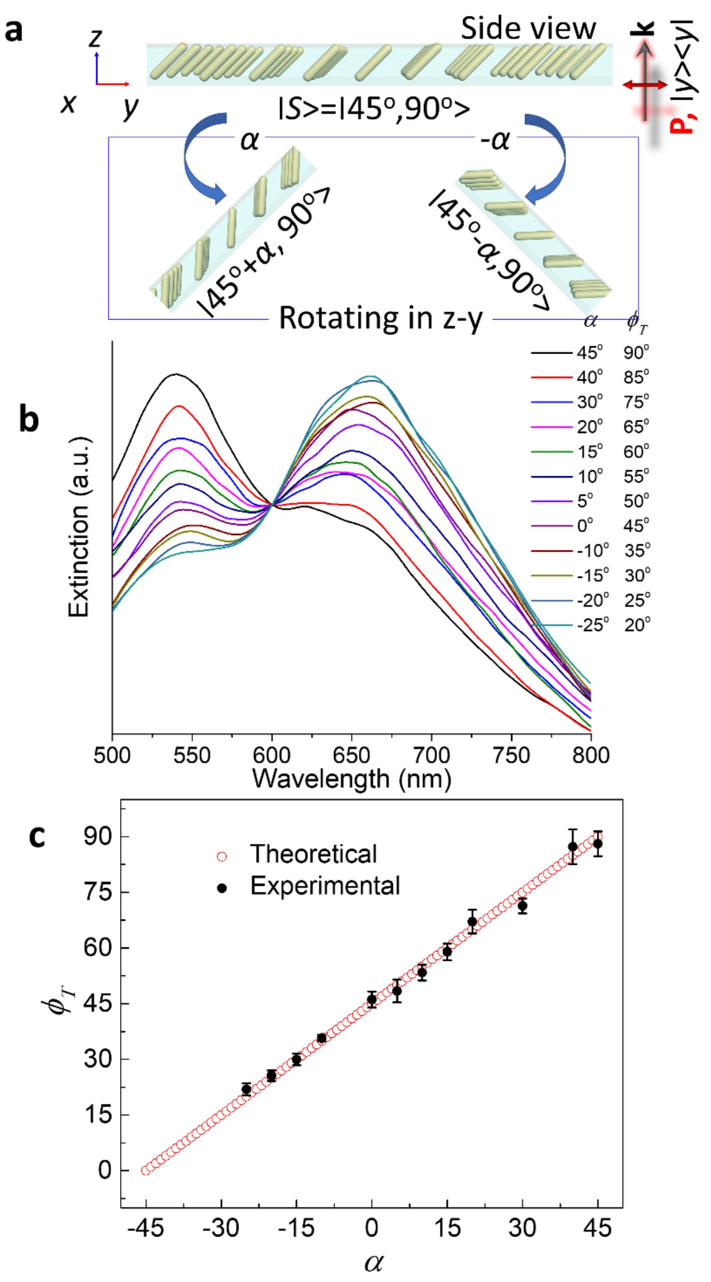

Supplementary Figure 19. Spectral analysis of plasmonic film upon rotating perturbation. a, Schematic illustration of excitation states of rotated plasmonic film. **b,** Extinction of plasmonic film under various rotating angles. The angles are positive if rotation is counterclockwise and negative for clockwise rotation. **c,** Linear dependence of phase angle of transverse extinction on rotational angle. Error bars represent the standard deviation from three experimental measurements.

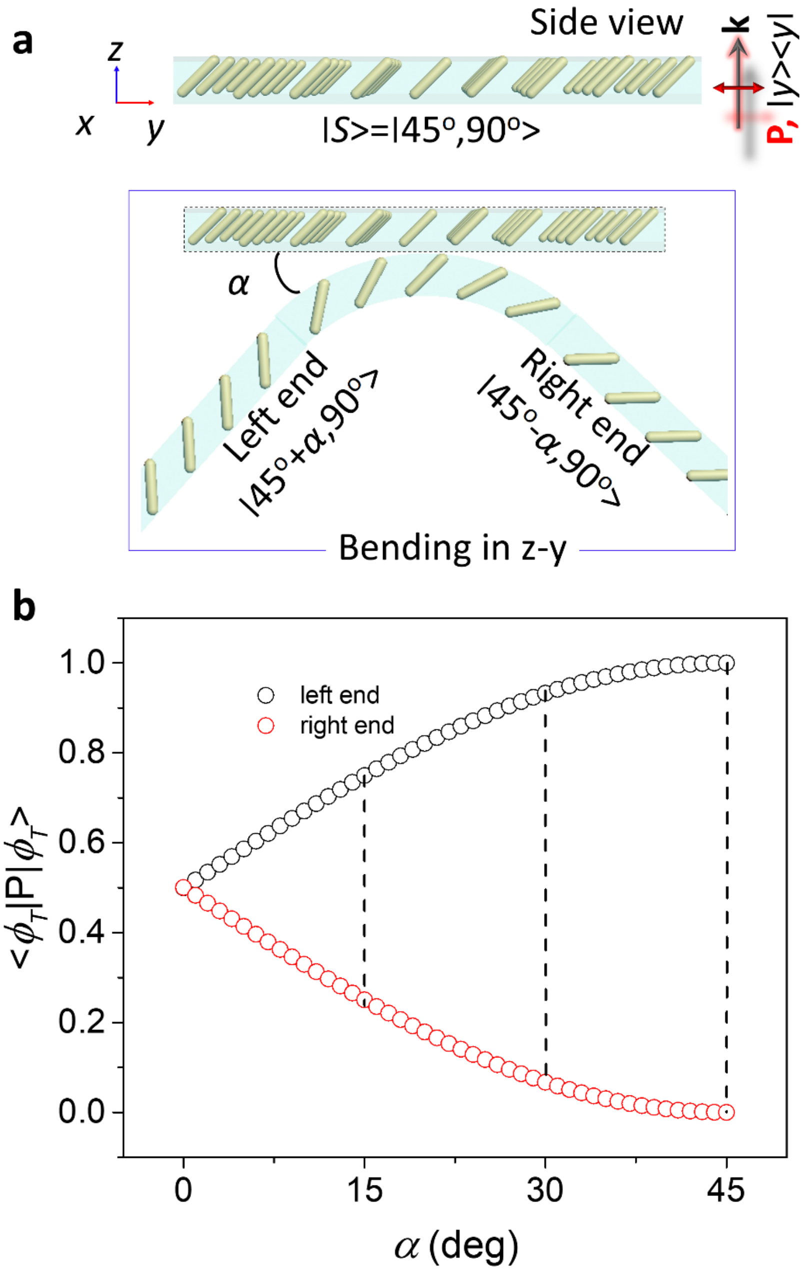

Supplementary Figure 20. Separation of excited states of plasmonic film upon bending. a, Schematic illustration of excitation states of bending plasmonic film. **b,** Analytical solution towards transverse excited states of two ends of plasmonic film upon bending axis.

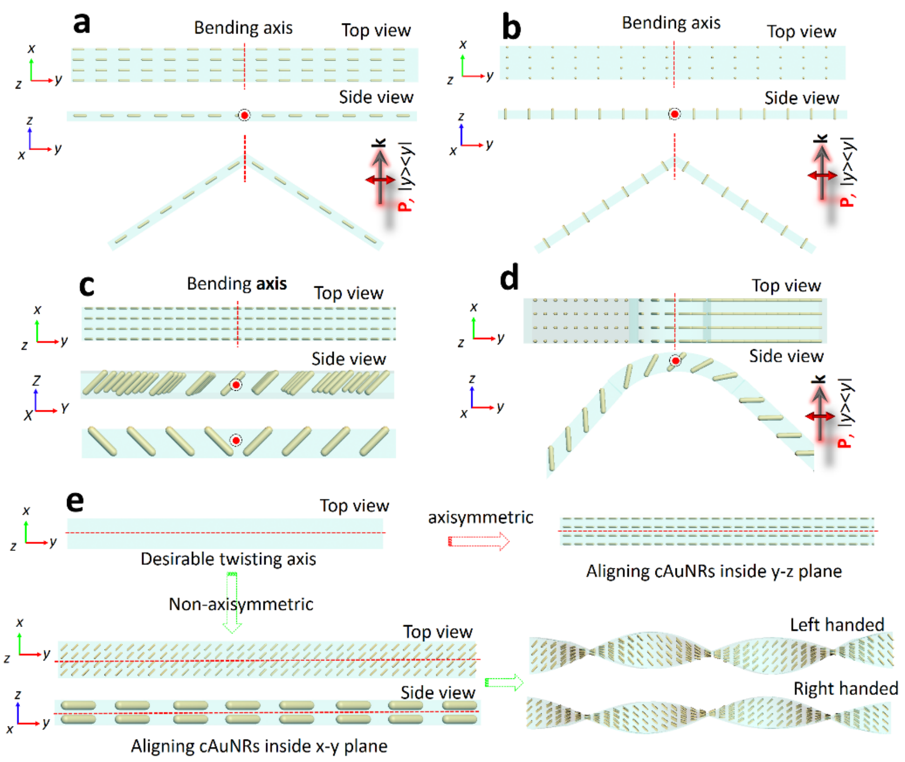

Supplementary Figure 21. Symmetry-breaking of orientation of cAuNRs along the active axis of mechanical motions induces the separation of excited states of plasmonic film upon mechanical motions. The alignment of cAuNRs in **(a)** and **(b)** is parallel and perpendicular to the film surfaces, respectively. The alignment of cAuNRs in both designs is axisymmetric about the bending axis, which induces same excitation states of cAuNRs in the two ends during bending. **c,** The 45° out-of-plane orientation of cAuNRs inside the plasmonic film. The specific alignment of cAuNRs breaks the axisymmetric orientation about the bending axis. For comparison, the axisymmetric alignment of cAuNRs is illustrated in the bottom panel. **d,** In the case of non-axisymmetric alignment, the excitation states of plasmonic resonance of cAuNRs at the two ends will be different, which will induce color contrast in response to bending. **e,** Predictive mechanochromic design for sensing twisting. Aligning cAuNRs along any directions inside y-z plane gives rise to axisymmetric arrangement, which will induce same plasmonic excitation under twisting. To break the symmetry, they need to be magnetically aligned inside x-y plane. For example, 45o inside x-y plane will induce different excitation states for cAuNRs in the neighbouring segments.

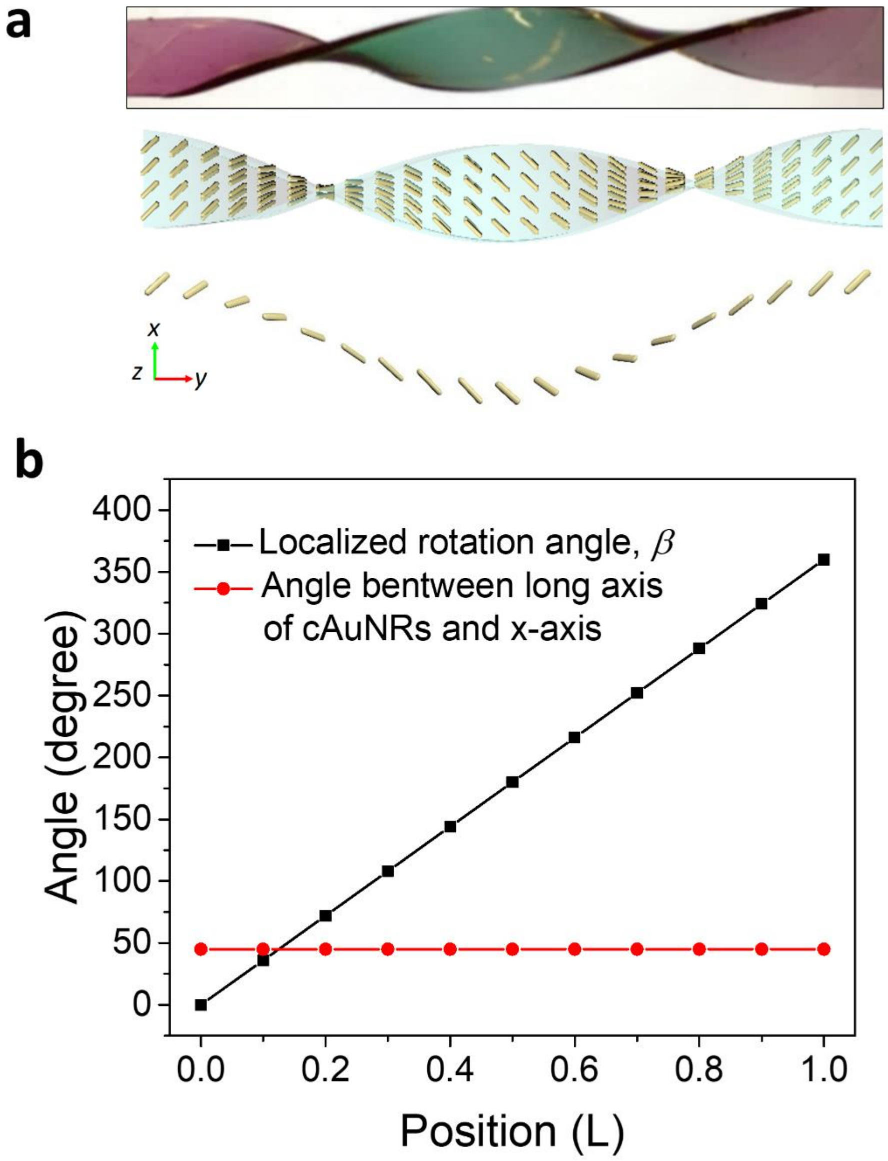

Supplementary Figure 22. a, Plasmonic film twisted by 360° and schematics mimicking the orientational distribution of cAuNRs at different locations. **b,** The angle between long axis of cAuNRs and y-axis remain constant as 45°, while the localized angle increases linearly as a function of position.

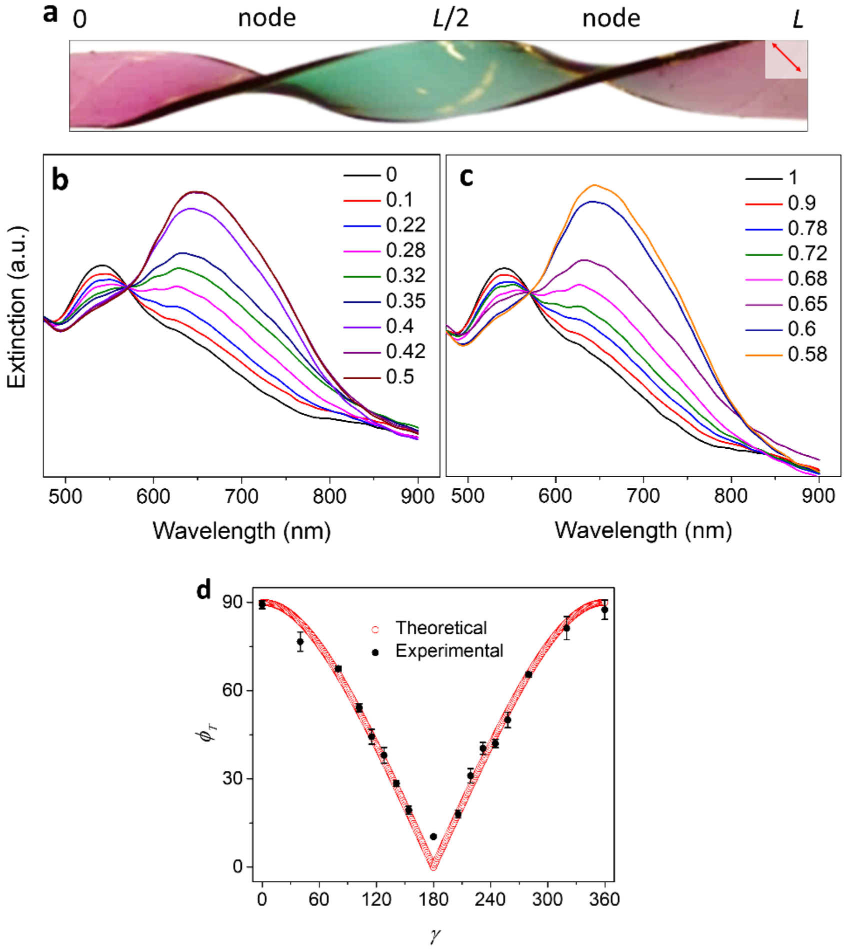

Supplementary Figure 23. Analyzing nonlinear twisting perturbation. a, Digital images of the plasmonic film upon twisting of 360°. The polarization direction is illustrated by red arrows. Space-resolved extinction spectra of left **(b)** and right end **(c)** of twisted plasmonic film. The indexes indicate the relative position where the corresponding spectrum was measured. **d,** Correlation between transverse phase angle, ϕ_T, and local rotation angle, γ. Error bars represent the standard deviation from three experimental measurements.

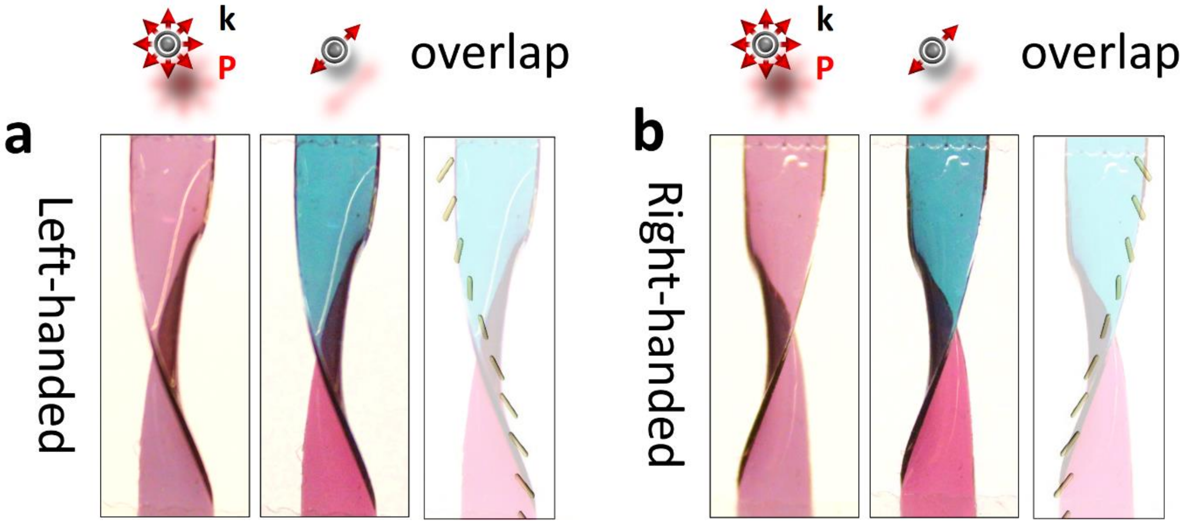

Supplementary Figure 24. Digital images of the (**a**) left-handed and (**b**) right-handed plasmonic film upon twisting of 180°. The polarization direction is illustrated by red arrows.

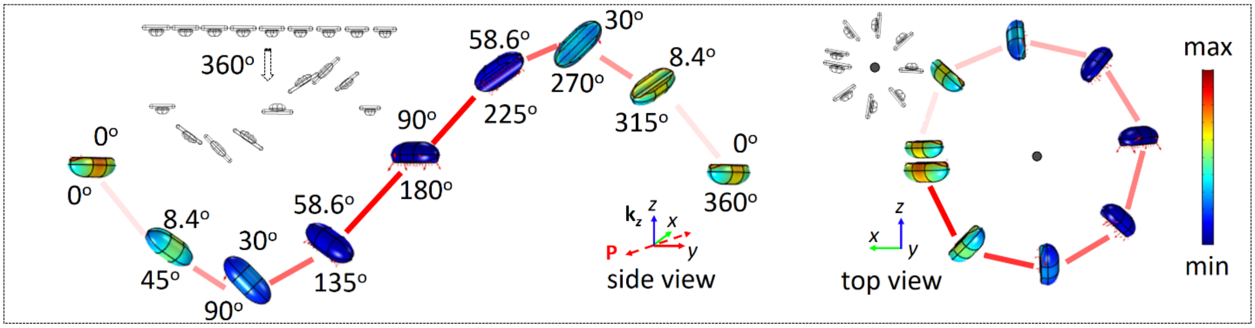

Supplementary Figure 25. Electric filed distribution (at 800 nm) and Poynting vectors (at 630 nm), *S*, on the surface of cAuNRs under a twisted configuration. In the side view, the numbers under and above the cAuNRs are γ and ϕ_T, respectively. The arrows and colors indicate the strength of transverse and longitudinal modes, respectively.

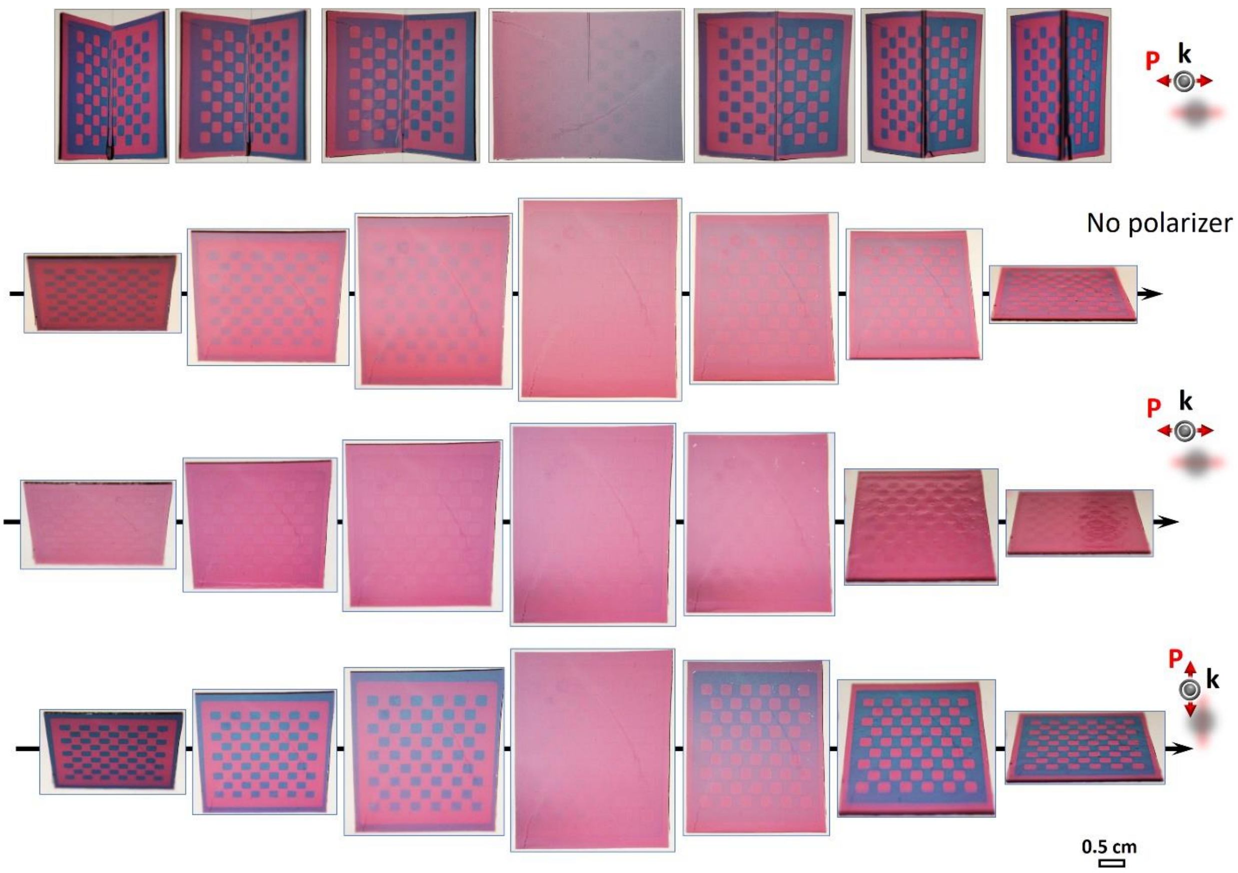

Supplementary Figure 26. Programmable mechanochromic response enabled by magnetic alignment. The same pattern can exhibit different mechanochromic response upon different mechanical perturbations. The film was subject to bending (first forwards and then backwards from left to right) in the top panel and rotating in the bottom three panels (also in a left-handed manner).

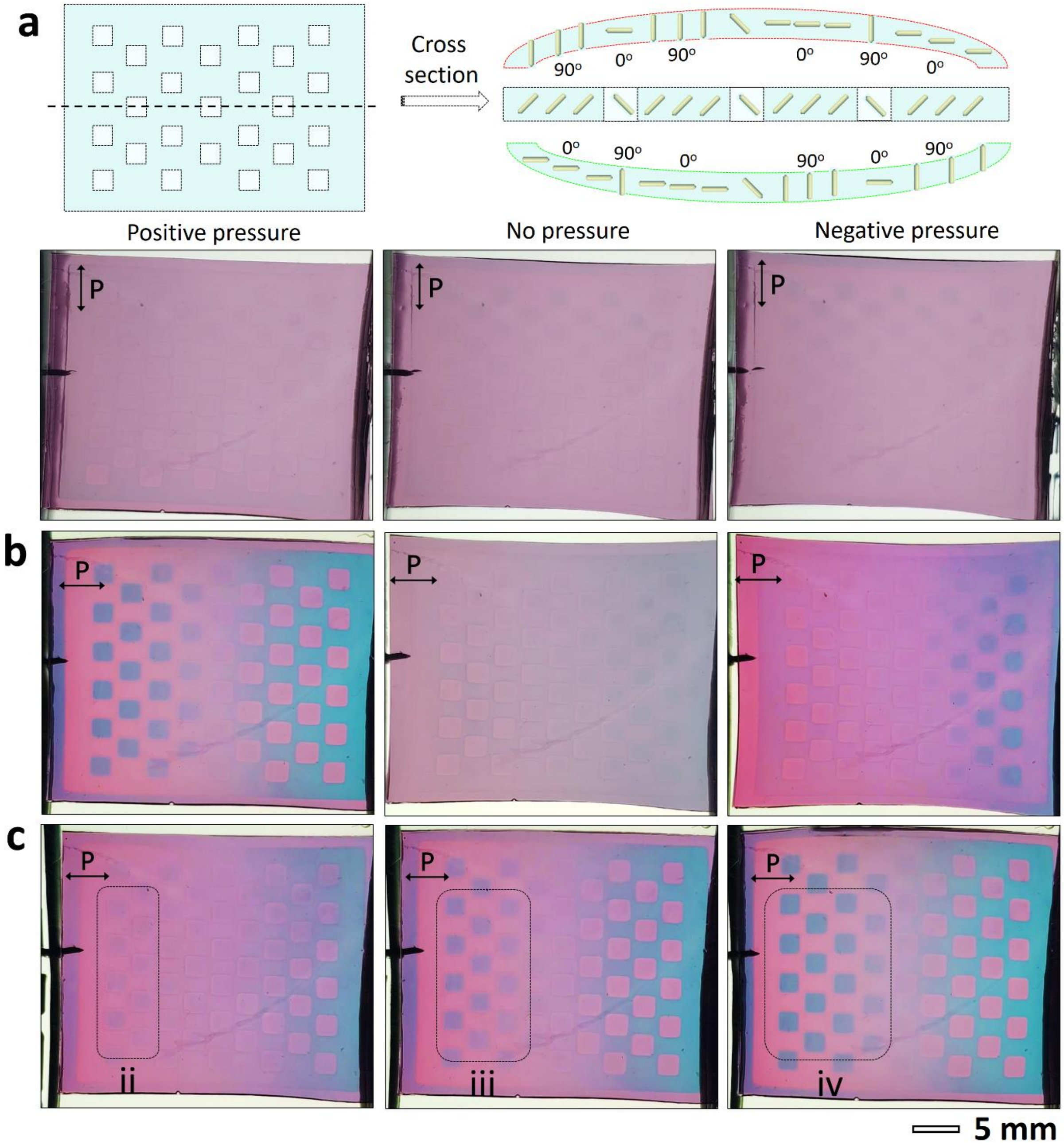

Supplementary Figure 27. Mechanochromic devices. In **(a)**, the orientation of cAuNRs was periodically arranged. The angles in the scheme indicates the transverse excitation angles. **b,** The mechanochromic response under negative and positive pressures. **c,** The mechanochromic response of the plasmonic films under different positive pressures.

Supplementary References

1. Li, Z. et al. Magnetic targeting enhanced theranostic strategy based on multimodal imaging for selective ablation of cancer. *Adv. Funct. Mater.* **24**, 2312-2321 (2014).
2. Gao, C., Zhang, Q., Lu, Z. & Yin, Y. Templated synthesis of metal nanorods in silica nanotubes. *J. Am. Chem. Soc.* **133**, 19706-19709 (2011).
3. Xu, W. et al. Chemical transformation of colloidal nanostructures with morphological preservation by surface-protection with capping ligands. *Nano Lett.* **17**, 2713-2718 (2017).
4. Demortiere, A. et al. Size-dependent properties of magnetic iron oxide nanocrystals. *Nanoscale* **3**, 225-232 (2011).
5. Lartigue, L. et al. Water-dispersible sugar-coated iron oxide nanoparticles. An evaluation of their relaxometric and magnetic hyperthermia properties. *J. Am. Chem. Soc.* **133**, 10459-10472 (2011).
6. McNab, T., Fox, R. & Boyle, A. Some magnetic properties of magnetite (Fe3O4) microcrystals. *J. Appl. Phys.* **39**, 5703-5711 (1968).
7. El Ghandoor, H., Zidan, H., Khalil, M.M. & Ismail, M. Synthesis and some physical properties of magnetite (Fe3O4) nanoparticles. *Int. J. Electrochem. Sci* **7**, 5734-5745 (2012).
8. Singh, G. et al. Self-assembly of magnetite nanocubes into helical superstructures. *Science* **345**, 1149-1153 (2014).
9. Huang, Y., Wu, L., Chen, X., Bai, P. & Kim, D.-H. Synthesis of anisotropic concave gold nanocuboids with distinctive plasmonic properties. *Chem. Mater.* **25**, 2470-2475 (2013).
10. Zhang, Q. et al. Faceted gold nanorods: Nanocuboids, convex nanocuboids, and concave nanocuboids. *Nano Lett.* **15**, 4161-4169 (2015).
11. Griffiths, D.J. Electrodynamics. *Introduction to Electrodynamics, 3rd ed., Prentice Hall, Upper Saddle River, New Jersey*, 301-306 (1999).
12. Wang, M., He, L., Xu, W., Wang, X. & Yin, Y. Magnetic assembly and field - tuning of ellipsoidal - nanoparticle - based colloidal photonic crystals. *Angew. Chem. Int. Ed.* **54**, 7077-7081 (2015).
13. Wang, M., He, L., Zorba, S. & Yin, Y. Magnetically actuated liquid crystals. *Nano Lett.* **14**, 3966-3971 (2014).
14. Liu, Q. et al. Self-alignment of plasmonic gold nanorods in reconfigurable anisotropic fluids for tunable bulk metamaterial applications. *Nano Lett.* **10**, 1347-1353 (2010).
15. Liu, Q., Yuan, Y. & Smalyukh, I.I. Electrically and optically tunable plasmonic guest–host liquid crystals with long-range ordered nanoparticles. *Nano Lett.* **14**, 4071-4077 (2014).
