# Supplementary material for: Coupling magnetic and plasmonic anisotropy in hybrid nanorods for mechanochromic responses
Source: Nat Commun. 2020 Jun 8;11:2883. doi: 10.1038/s41467-020-16678-8 (PMC7280256; doi:10.1038/s41467-020-16678-8)
Supplement: Supplementary file 3 — Description of Additional Supplementary Files [file 41467_2020_16678_MOESM3_ESM.pdf]

Description of Additional Supplementary Files

File Name: Supplementary Movie 1
Description: Color change in colloidal dispersion of short cAuNRs

File Name: Supplementary Movie 2
Description: Color change in colloidal dispersion of long cAuNRs

File Name: Supplementary Movie 3
Description: Mechanochromic response of plasmonic films to stretching

File Name: Supplementary Movie 4
Description: Mechanochromic response of plasmonic films to linear bending

File Name: Supplementary Movie 5
Description: Mechanochromic response of plasmonic films to nonlinear twisting

File Name: Supplementary Movie 6
Description: Polarization-dependent coloration of plasmonic films

File Name: Supplementary Movie 7
Description: Mechanochromic devices
