# Supplementary material for: Coupling magnetic and plasmonic anisotropy in hybrid nanorods for mechanochromic responses
Source: Nat Commun. 2020 Jun 8;11:2883. doi: 10.1038/s41467-020-16678-8 (PMC7280256; doi:10.1038/s41467-020-16678-8)
Supplement: Supplementary file 2 — Peer Review File [file 41467_2020_16678_MOESM2_ESM.pdf]

Editorial Note: This manuscript has been previously reviewed at another journal that is not operating a transparent peer review scheme. The manuscript was considered suitable for publication without further review at *Nature Communications*.
